# Preformulation Study of Controlled-Release Galantamine Matrix Tablets Containing Polyethylene Oxide, Hydroxypropyl Methylcellulose, and Ethylcellulose

**DOI:** 10.3390/pharmaceutics17091139

**Published:** 2025-08-30

**Authors:** Andres C. Arana-Linares, Paola A. Caicedo, María Francisca Villegas-Torres, Andrés F. González-Barrios, Natalie Cortes, Edison H. Osorio, Constain H. Salamanca, Alvaro Barrera-Ocampo

**Affiliations:** 1 Grupo Natura, Departamento de Ciencias Farmacéuticas y Químicas, Facultad Barberi de Ingeniería, Diseño y Ciencias Aplicadas, Universidad Icesi, Calle 18 No. 122-135, Cali 760031, Colombia; andres.arana1@u.icesi.edu.co; 2 Grupo QBAB, Instituto de Ciencias Aplicadas, Facultad de Ingeniería, Universidad Autónoma de Chile, El Llano Subercaseaux 2801, San Miguel, Santiago 8910060, Chile; 3 Grupo Natura, Departamento de Ciencias Biológicas, Bioprocesos y Biotecnología, Facultad Barberi de Ingeniería, Diseño y Ciencias Aplicadas, Universidad Icesi, Calle 18 No. 122-135, Cali 760031, Colombia; pacaicedo@icesi.edu.co; 4 Centro de Investigaciones Microbiológicas (CIMIC), Department of Biological Sciences, Universidad de los Andes, Bogotá 111711, Colombia; m.f.villegastorres@uniandes.edu.co; 5 Grupo de Diseño de Productos y Procesos (GDPP), Department of Chemical and Food Engineering, Universidad de los Andes, Cra 1 Nº 18A-12, Bogotá 111711, Colombia; andgonza@uniandes.edu.co; 6 Grupo de Investigación NATURATU, Faculty of Natural Sciences and Mathematics, Universidad de Ibagué, Ibagué 730001, Colombia; natalie.cortesre@amigo.edu.co (N.C.); edison.osoriolo@amigo.edu.co (E.H.O.); 7 Departamento de Ciencias Básicas, Universidad Católica Luis Amigó, Transversal 51A No. 67B-90, Medellín 050034, Colombia; 8 Departamento de Farmacia, Facultad de Ciencias Farmacéuticas y Alimentarias, Universidad de Antioquia, Calle 70 No. 52-21, Medellín 050010, Colombia; 9 Grupo de Investigación Ciencia de Materiales Avanzados, Departamento de Química, Facultad de Ciencias, Universidad Nacional de Colombia Sede Medellín, Cra. 65 #59a-110, Medellín 050034, Colombia; 10 Grupo de Investigación Cecoltec, Cecoltec Services SAS, Medellín 050034, Colombia

**Keywords:** compaction behavior, controlled-release matrix tablets, galantamine formulation, polymeric drug delivery systems, granulometric analysis

## Abstract

**Background/Objectives**: The rational design of modified-release matrix tablets requires a thorough understanding of granulometric analysis, compaction behavior, and drug release profile. In this study, we evaluated the physicochemical, granulometric, and mechanical properties of hydroxypropyl methylcellulose, polyethylene oxide, and ethylcellulose in galantamine matrix formulations. **Methods**: Spectroscopic (FTIR) and thermal (DSC) analyses demonstrated drug–polymer compatibility. We assessed flowability, cohesion, and aeration behavior through granulometric analysis and applied compressibility models (Kawakita, Heckel, Leuenberger) to characterize deformation mechanisms. **Results**: Hydroxypropyl methylcellulose showed superior compactability (T_max_ = 4.61 MPa) and sustained drug release (85.4% at 12 h, DE% = 62.2%), while polyethylene oxide enabled gradual erosion and consistent delivery (88.7% at 12 h, DE% = 57.5%). In contrast, ethylcellulose exhibited high cohesiveness but poor matrix integrity, leading to premature drug release (76.6% at 1 h, DE% = 73.7%). Only hydroxypropyl methylcellulose and polyethylene oxide formulations met USP criteria. **Conclusions**: These results demonstrate that polymer selection critically influences powder behavior and matrix performance, underscoring the need for integrated granulometric and mechanical evaluation in the development of robust controlled-release systems.

## 1. Introduction

Galantamine (GAL) is an acetylcholinesterase inhibitor and an allosteric modulator of nicotinic receptors [1]. GAL is a weak base with an ionization constant (pKa = 8.2), which is due to the presence of the azepine moiety. It displays moderate lipophilicity, as indicated by a partition coefficient (n-octanol/buffer, pH 12.0) of 1.1, and an aqueous solubility of 31 mg/mL at pH 6.0 [2]. Based on these physicochemical properties, GAL is classified as a Biopharmaceutics Classification System (BCS) Class I drug, characterized by high solubility and high permeability. Its clinical use was approved in 2000 by the European Union and in 2001 by the United States for the symptomatic treatment of Alzheimer’s disease [3]. It is still used today because it has demonstrated benefits in cognitive function, overall performance, and a lower incidence of neuropsychiatric symptoms [4,5,6,7]. GAL has an elimination half-life of approximately 6.8 h, requiring twice-daily dosing [8]. This dosing regimen may contribute to a higher incidence of adverse effects, including nausea, vomiting, anorexia, weight loss, dizziness, and depression [9,10]. Additionally, GAL-based medications are often not manufactured in developing countries, such as Colombia. Instead, they are imported and marketed by multinational companies such as JANSSEN CILAG S.A., resulting in high costs for the healthcare system and limited patient access. This limitation, combined with the scarcity of studies focused on the development of modified-release formulations with GAL, highlights the need for continued research in this area so that the design of a locally manufactured tablet formulation can be consolidated as both a pharmaceutically and socially relevant alternative.

Modified-release tablets are among the most used solid dosage forms for the gradual and controlled release of a drug over a prolonged period [11,12,13,14,15,16,17,18]. These systems offer several advantages over immediate-release tablets, such as reduced dosing frequency, lower total drug load, and minimized fluctuations in the plasma concentration of the active pharmaceutical ingredient (API). These benefits are associated with a lower incidence of adverse events, improved therapeutic adherence and patient quality of life, and a reduction in healthcare-related costs [19,20,21,22]. Polymeric matrix systems are widely employed in the design of modified-release tablets due to their ability to achieve desirable drug release profiles, ease of manufacturing, cost-effectiveness, and broad regulatory acceptance [23,24,25,26,27,28,29,30]. Among the various technologies available, the polymeric matrix tablet was selected in this study because it offers robust performance, enhanced stability, and compatibility with direct compression, one of the simplest and most economical manufacturing methods [31]. In contrast, multiparticulate systems such as pellets or granules typically require more complex processes, specialized equipment, and higher production costs [32,33]. These advantages make matrix tablets a more suitable and feasible option for local pharmaceutical development and potential technology transfer [34,35].

The excipients used in the production of polymeric matrix tablets are generally classified as soluble or hydrophilic, such as hydroxypropyl methylcellulose (HPMC) and polyethylene oxide (PEO), and insoluble or hydrophobic matrix formers, such as ethyl cellulose (EC) [36,37,38,39,40,41,42,43,44,45,46,47,48,49]. HPMC is a cellulose derivative composed mainly of glucose chains substituted with methyl and hydroxypropyl groups [39], while PEO is produced through the heterogeneous catalytic polymerization of ethylene oxide monomers [47,48,50]. Hydrophilic polymeric matrices, upon contact with water or physiological fluids, hydrate, swell, and form a highly concentrated polymer solution (gel layer) that modulates the release mechanism of the API either by diffusion through the gel or by erosion of the polymer matrix [51,52]. On the other hand, EC is a partially O-ethylated cellulose ether that forms hydrophobic polymeric matrices. These matrices remain intact during drug release and are excreted as empty scaffolds [53,54]. In this context, these three polymers were selected based on their local availability, well-established safety profile in pharmaceutical products, and their ability to effectively modulate drug release kinetics. Although other functional excipients are available—such as sodium carboxymethylcellulose or acrylic polymers like Eudragit^®^—the choice of HPMC, PEO, and EC allows for coverage of a representative range of release mechanisms (diffusion, erosion, and hydrophobic barrier) without the need for complex manufacturing processes [52,55,56,57].

Therefore, the objective of this study was to evaluate the physicochemical and granulometric properties of HPMC, PEO, and EC, and to determine their influence on the compressibility and release behavior of galantamine in matrix tablets. The underlying hypothesis is that the selection of appropriate polymers with favorable flow and compaction characteristics can enable the development of a robust modified-release system for GAL. The results of this study are expected to provide a scientific basis for the formulation of a cost-effective and locally manufacturable oral dosage form that could improve access to this medication in settings with limited availability.

## 2. Materials and Methods

### 2.1. Materials

GAL hydrobromide (99.8% as C_17_H_21_NO_3_·HBr) was purchased from Aurobindo Pharma Ltd., Hyderabad, Telangana, India. Polyethylene oxide (POLYOX^TM^ WSR N12K LEO, 95.0–100.0% PEO), hydroxypropyl methylcellulose (METHOCEL^TM^ K15M, 85.0–99.0% HPMC), ethylcellulose (ETHOCEL^TM^ Standard 10 FP, 98.0–100.0% EC), and Partially Pregelatinized Maize Starch (Starch^®^ 1500) were purchased from Colorcon^®^, Harleysville, PA, USA. Spray-dried lactose monohydrate (Spray Dried Fast Flo^®^ 316) was purchased from Kerry, Tralee, Kerry, Ireland. Colloidal silicon dioxide (Aerosil 200) was purchased from Evonik Operations GmbH, Essen, Ruhr, Germany. Magnesium stearate was purchased from Anhui Sunhere Pharma, Huainan, Anhui, China. Other reagents, including KOH, KCl, KH_2_PO_4_, and K_2_HPO_4_ were obtained from Merck KGaA, Darmstadt, Hesse, Germany. Type II water was obtained from a Millipore Elix essential purification system (Merck KGaA, Darmstadt, Hesse, Germany).

### 2.2. Preparation of Powder Mixtures

For the preparation of the powder blends, the excipients and active ingredient were sieved using mesh screens of different sizes: No. 60 (250 µm) for PEO, HPMC EC, and spray-dried lactose monohydrate; No. 30 (595 µm) for GAL and Starch^®^ 1500; and No. 20 (841 µm) for magnesium stearate. The components were then mixed in a multi-directional powder blender for 5 min at 20 rpm. The proportion of the active ingredient and each excipient in the three formulations is detailed in Table 1.

### 2.3. Evaluation of Excipient Compatibility Through Thermal and Spectroscopic Analysis

Structural characterization of the pure formulation ingredients and binary mixtures (1:10 drug:polymer) of GAL hydrobromide with controlled-release polymers was carried out using an FT-IR spectrometer (Nicolet 6700, ThermoScientific, Waltham, MA, USA). The spectra were recorded in the frequency range of 4000 to 400 cm^−1^. Furthermore, thermal analysis was performed on a Q2000 differential scanning calorimeter (DSC; TA Instruments, New Castle, DE, USA) calibrated with indium (T_m_ = 155.78 °C ∆H_m_ = 28.71 J/g). Three modulated heating–cooling cycles were conducted from 25 °C (298.15 K) to 400 °C (673.15 K) at a heating rate of 5.0 °C/min, under a constant flow of high-purity nitrogen gas (grade 5.0) at 50 mL/min.

### 2.4. Physical Analysis of Powder Excipients and Formulations

#### 2.4.1. Particle Size and Size Distribution

The particle size and size distribution of the powder excipients and tablet formulations were determined using static light scattering (SLS) with a MasterSizer 3000 (Malvern Panalytical, Malvern, Worcestershire, UK) equipped with a helium–neon laser operating at a wavelength of 632.8 nm.

#### 2.4.2. Granulometric Characterization Based on Flowability Indices and Porosity

The flowability and cohesiveness of the powders were evaluated prior to compression by assessing their flow behavior, packing ability, and degree of cohesiveness. These properties were quantified using the Carr Index, Hausner Ratio, and porosity, which characterize the powders in their free-flowing state and provide insight into their behavior during subsequent processing [58,59]. The Carr Index (%) assesses the cohesiveness of the material and is calculated as:

(1) $$Carr\;Index\left(\%\right)=\left(\frac{\rho_{Tapped}-\rho_{Bulk}}{\rho_{Tapped}}\right)\times 100$$

The Hausner Ratio serves as an indicator of flowability, as follows:

(2) $$Hausner\;ratio=\left(\frac{\rho_{Tapped}}{\rho_{Bulk}}\right)$$

Porosity (%) is determined by comparing the bulk and true densities:

(3) $$\varepsilon \left(\%\right)=\left(1-\frac{\rho_{bulk}}{\rho_{true}}\right)\times 100$$

The true densities were determined with an AccupycII 1340 helium displacement pycnometer (Micromeritics Corp., Norcross, GA, USA). Bulk and tapped densities were measured using a VanKel 50-1000 Tap Density Tester (VanKel Industries, Cary, NC, USA).

### 2.5. Compressibility, Compactability, and Tabletability Analysis

#### 2.5.1. Powder Compression

Approximately 200 mg of powder from Formulations 1, 2, and 3 were compressed using a Model C hydraulic press (Carver Press, Menomonee Falls, WI, USA) equipped with compression pressure control. Compression was performed at pressures ranging from 5 to 50 MPa, using a 6.5 mm diameter flat-faced punch and die, with a dwell time of 10 s. The upper punch was fitted with an LCGD-10K load cell (Omega Engineering Inc., Stamford, CT, USA) with a range of 0 to 10,000 psi (0 to 68.9 MPa), connected to a DP25B-S strain indicator (Omega Engineering Inc., Stamford, CT, USA) for real-time monitoring of applied pressure.

#### 2.5.2. Kawakita Model

The Kawakita and Lüdde model [60] was used to establish a correlation between powder densification and its physical properties through the study of tapping compression, measuring the reduction in powder bed volume after applying 400 (*N*) tapping cycles.

(4) $$\frac{N}{C_{T}}=\frac{N}{a_{T}}+\frac{1}{a_{T}b_{T}},\;C_{T}=\frac{V_{0}-V_{t}}{V_{0}}$$

where *V*_0_ corresponds to the initial volume of the powder bed, *V_t_* denotes the volume after *N* tapping cycles. The constant *a_T_* is indicative of Carr’s Index, and *b_T_* is related to the cohesiveness of the powder [61].

#### 2.5.3. Heckel Model

The Heckel model delves into the deformation mechanism of powdered materials during compression, differentiating between plastic deformation and fragmentation and their impact on tablet consolidation. This model evaluates the plastic deformation of powder under pressure and is defined according to:

(5) $$\mathrm{Ln}\left(\frac{1}{1-D}\right)=K\times P+A,D=\frac{\rho_{bulk}}{\rho_{tap}},\;\varepsilon =1-D$$

where D is the relative compact density (solid fraction) at pressure P, ε is the compact porosity, K is the compressibility constant, which indicates whether the material deforms plastically or fragments under the application of compression, and A is the intercept, define as the powder densification by matrix filling and particle rearrangement before deformation and bonding of discrete particles occurs. The slope of the linear segment of the plot is inversely related to the yield pressure ($P_{y}$) or yield stress, which is a measure of the plasticity of the compressed material [62].

#### 2.5.4. Leuenberger Model

The radial tensile strength (TS) values were obtained according to the Fell and Newton equation from the breaking force given by the load-deflection curves [63] as follows:

(6) $$TS(MPa)=\frac{2F}{\pi \times D\times h}$$

where F is the breaking force necessary to break the tablet into two halves, and which is obtained by means of a durometer HDT-400 (Logan Instruments Corp., Somerset, NJ, USA, D is the diameter of the tablet (mm), and h is the height or thickness of the tablet (mm), which were measured with a digital caliper.

The radial tensile strength data versus the product of the solid fraction and the compression pressure were fitted according to the Leuenberger model, which combines the previous models to evaluate the quality of the final tablet, integrating the formulated system’s compressibility, compactibility, and mechanical strength to ensure its structural stability. This structure ensures a clear progression from the initial characterization of the powder to its performance in tablet manufacturing. These models combine compressibility and compactibility to evaluate the tablet’s mechanical strength, and is defined according to:

(7) $$TS=T_{max}\times (1-e^{\left(-\gamma_{c}*P*\rho_{r}\right)})$$

where *T_max_* is the theoretical tensile strength at infinite compression pressure, *γ_c_* is the compression susceptibility parameter (MPa^−1^), *ρ_r_* is the compact relative density, and *P* is the compression pressure.

### 2.6. Preparation and Characterization of Controlled-Release Matrix Tablets

**Figure 1:**
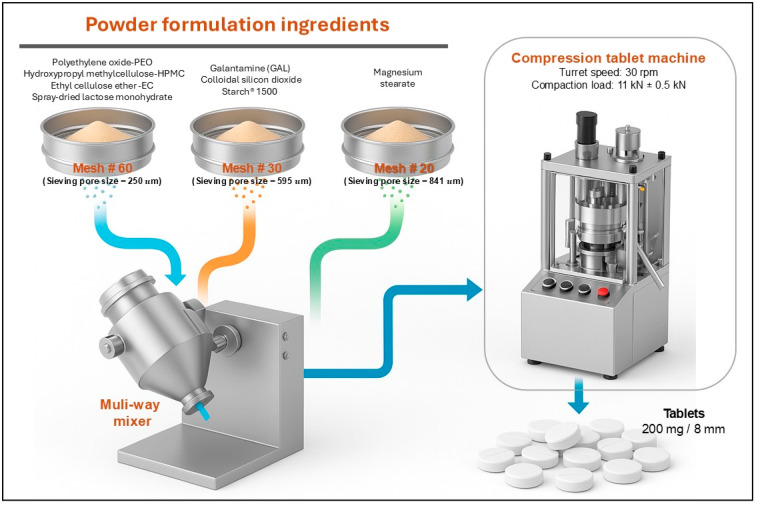
Schematic of GAL controlled-release tablet preparation. Excipients and GAL (sieved to 250–841 µm) were blended in a multi-way mixer, then compressed at 30 rpm and 11 ± 0.5 kN to yield 200 mg, 8 mm tablets.

GAL controlled-release matrix tablets were prepared by direct compression, as shown in Figure 1. Each tablet had a total weight of 200 mg, and the formulations were compressed using a Picolla-Riva tablet press (Riva S.A., Buenos Aires, Argentina).

Three groups of excipients and active ingredients—sieved through mesh #60 (250 µm), mesh #30 (595 µm), and mesh #20 (841 µm)—were introduced into a multi-way powder mixer. The components include PEO, HPMC, spray-dried lactose monohydrate, GAL, colloidal silicon dioxide, starch^®^ 1500, and magnesium stearate. After thorough blending, the uniform powder mixture was fed into a compression tablet machine operating at a turret speed of 30 rpm and a compaction load of 11 kN ± 0.5 kN. TSM (IPT)-B upper and lower punches of 8 mm diameter were used, and the process produced tablets with a mass of 200 mg.

#### 2.6.1. Tablet Physical Characterization

Average tablet weight, hardness, friability, and disintegration were tested. The tablets were weighed with an analytical scale PRACTUM22418 (Sartorius Lab Instruments, Gottingen, Gottingen, Germany). A durometer HDT-400 breaking force tester (Logan Instruments Corp., Somerset, NJ, USA) was used to determine the tablet breaking strength, whilst the diameter measurement was carried out using a caliper. Friability tests were performed according to USP Chapter 1216 with a FAB-2S (Logan Instruments Corp., Somerset, NJ, USA). A disintegration test was performed on six tablets with a USP DST-3 (Logan Instruments Corp., Somerset, NJ, USA) apparatus following the test USP Chapter 701. PBS buffer solution pH 6.5 (50 mM) was used as a medium.

#### 2.6.2. In Vitro Dissolution Tests

HPLC analyses were carried out using a Hitachi Lachrom Elite model chromatograph (Hitachi High-Technologies Corporation, Shinjuku, Tokio, Japan). The diode-array detector was set at wavelength 210 nm and peak areas were integrated automatically by computer using EZ Chrom Elite Software (ver. 3.3.2 SP1). Separation was achieved using a KINETEX C18A–100 A column (150 × 4.6 mm; 2.6 µm; Phenomenex, Torrance, CA, USA). The mobile phase was made up of buffer solution pH6.5 50mM and methanol applied at a flow rate of 0.5 mL/min, column temperature 25 °C, and 10 µL portions were injected into the liquid chromatography system.

A Vision^®^ G2 Classic 6 dissolution equipment (Hanson Equipment Co., Lockhart, TX, USA) was used for the in vitro release test. The assay was performed for 12 h according to the specifications of the USP monograph Galantamine, Extended-Release Capsules [64] and the dissolution medium comprising 900 mL of PBS buffer pH 6.5 (50 mM) maintained at 37 °C  ±  0.1 °C and stirred at a rpm of 50. Aliquots, 5 mL each, were withdrawn at the time intervals of 1, 4 and 12 h during the studies and replaced by an equal amount of buffer. The concentration of GAL was determined as follows:

(8) $$C_{C_{17}H_{21}{NO}_{3}}=\frac{R_{u}}{R_{s}}\times C_{s}\times V\times \frac{1}{L}\times \frac{M_{r1}}{M_{r2}}\times 100$$

where *Ru* and *Rs* represent the peak responses of the sample and standard solutions, respectively; *Cs* is the concentration of GAL in the standard solution; *V* is the volume of the dissolution medium; *L* is the amount of GAL per tablet; and *Mr*_1_ and *Mr*_2_ are the molecular weights of GAL (287.35 g/mol) and GAL hydrobromide (368.27 g/mol), respectively.

The data obtained from the in vitro dissolution profiles are reported as the average dissolution efficiency (*DE*%) of the tablet [65]. This parameter is defined as the area under the dissolution curve (*AUC*) recorded at a particular time in relation to the rectangular area (*R*) described by 100% of dissolution at the same time and where *y* is the dissolved drug percentage at time *t*. The efficiency of the solution can be calculated from:

(9) $$DE=\frac{AUC}{R}\times 100=\frac{\int_{0}^{t}y\times dt}{y_{100}\times t}\times 100$$

### 2.7. Data Analysis

All data were obtained in triplicate and are reported as the mean value with the corresponding standard deviation. Likewise, the data were treated using OriginPro, version 2024 (Northampton, MA, USA), and GraphPad Prism, version 8.0.2 (Northampton, MA, USA), software. Nonlinear curve fitting for the Leuenberger Model was performed using an iterative algorithm based on Orthogonal Distance Regression.

## 3. Results and Discussion

### 3.1. Evaluation of Excipient Compatibility Through Thermal and Spectroscopic Analysis

#### 3.1.1. Pure Formulation Ingredients

**Figure 2:**
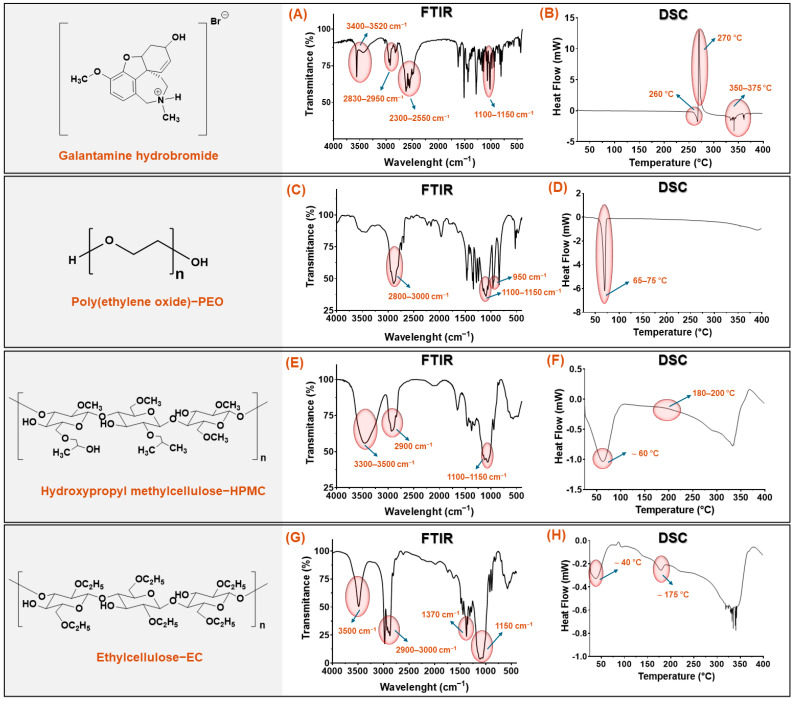
FTIR spectra (**A**,**C**,**E**,**G**) and DSC thermograms (**B**,**D**,**F**,**H**) of GAL, PEO, HPMC, and EC. FTIR confirmed characteristic functional groups, while DSC showed melting, glass transition, and decomposition events, highlighting compound identity and thermal behavior for formulation.

The spectroscopic (FTIR) and thermal (DSC) characterization results for pure GAL hydrobromide and the controlled-release polymers are shown in Figure 2.

FTIR spectra (Figure 2A,C,E,G) display characteristic functional group vibrations: broad O–H/N–H stretching (3400–3520 cm^−1^), C–H stretching (2800–3000 cm^−1^), and other key peaks such as C–O, C–O–C, and C–N bands between 1100 and 1370 cm^−1^, specific to each compound. DSC thermograms (Figure 2B,D,F,H) show thermal transitions: melting points, glass transition temperatures, and decomposition events. GAL shows a sharp melting peak at ~270 °C, while the polymers exhibit broad endothermic transitions reflecting their semi-crystalline or amorphous nature. These analyses confirm compound identity and thermal behavior relevant for tablet formulation.

For GAL hydrobromide (Figure 2A), the FTIR spectrum displayed characteristic bands consistent with its functional structure. The region between 3400 and 3520 cm^−1^ showed two overlapping contributions: a broad, low-intensity band near 3400 cm^−1^, attributed to the O–H stretching of the aliphatic hydroxyl group in the cyclohexenol moiety, and a sharper, more intense band around 3520 cm^−1^, corresponding to the N–H stretching of the tertiary ammonium cation (R_3_N^+^–H) [66]. Additional signals were observed between 2950 and 2830 cm^−1^ corresponding to aliphatic C–H stretching, along with a weak band between 2300 and 2550 cm^−1^ attributed to overtones of ionized species, signals between 1450 and 1600 cm^−1^ assigned to C–H deformations and C=C stretching, and a strong band between 1100 and 1150 cm^−1^ related to C–O–C stretching. The thermal analysis (Figure 2B) showed a well-defined endothermic transition at 260 °C, attributed to melting, followed immediately by an exothermic signal at 270 °C, associated with the onset of thermal degradation. This proximity between melting and degradation has been extensively reported for organic salts with rigid crystalline structures [67] and specifically described for GAL hydrobromide [68], where an endothermic event at approximately 270 °C is followed by degradation at ~278–280 °C. Endothermic events were also identified between 350 and 375 °C, attributed to secondary thermal processes such as melting or breakdown of degradation products.

The polymeric excipients used as release matrices showed characteristic spectroscopic and thermal signatures. In the case of polymer PEO, the FTIR spectrum (Figure 2C) displayed C–H stretching bands between 2800 and 3000 cm^−1^, a strong band between 1100 and 1150 cm^−1^ corresponding to asymmetric C–O–C stretching of the polyether backbone, and a secondary band around 950 cm^−1^ associated with CH_2_ skeletal vibrations [69]. Its DSC thermogram (Figure 2D) evidenced an endothermic peak at 68.5 °C, corresponding to its melting point, which matches the 65–70 °C range reported for high molecular weight PEO [70].

In the case of polymer HPMC, the FTIR spectrum (Figure 2E) exhibited a broad O–H stretching band between 3300 and 3500 cm^−1^, C–H stretching around 2900 cm^−1^, and a strong C–O–C band at 1110 cm^−1^ [71]. Its DSC thermogram (Figure 2F) displayed an initial endothermic signal at 61.2 °C, associated with moisture loss, followed by a glass transition between 185 and 195 °C, which agrees with the Tg range of 190–200 °C reported for HPMC E15 [72].

Finally, the FTIR spectrum of polymer EC (Figure 2G) showed bands between 2900 and 3000 cm^−1^ (C–H stretching), a signal at 1370 cm^−1^ (C–H bending), and a prominent band at 1150 cm^−1^ (C–O–C stretching), along with a weak absorption around 3500 cm^−1^ attributed to residual –OH groups [73]. Its DSC thermogram (Figure 2H) revealed a weak signal at ~38.7 °C related to segmental mobility and an endothermic signal between 172 and 178 °C, consistent with the reported melting point for Ethyl cellulose 100 [74]. Overall, the correlation between the experimental profiles and literature-reported values confirms the structural identity and thermal stability of the materials evaluated, providing a robust foundation for assessing their performance and interactions in multicomponent systems.

#### 3.1.2. Binary Mixtures of GAL Hydrobromide and Controlled-Release Polymers

**Figure 3:**
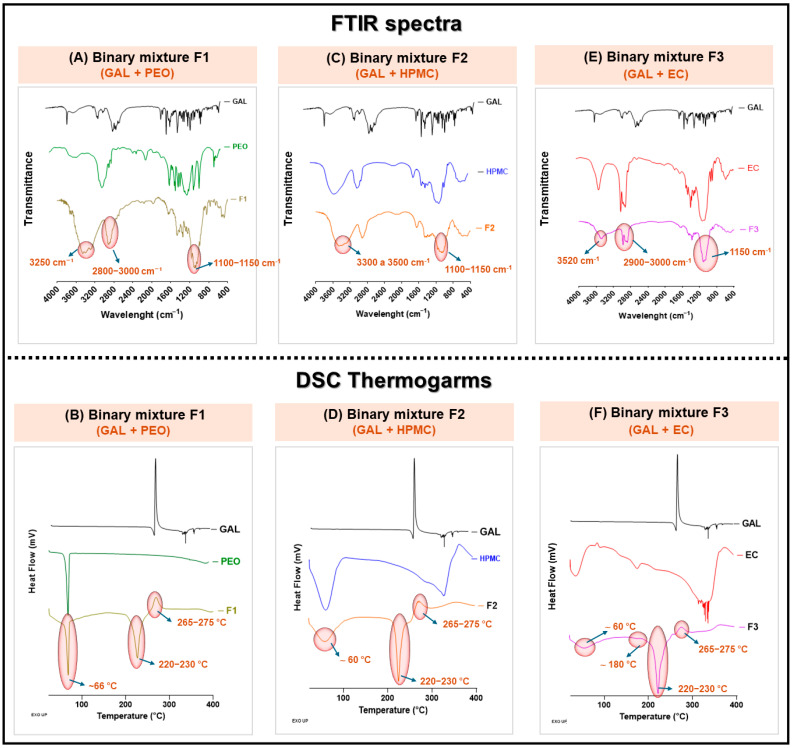
FTIR spectra (**A**,**C**,**E**) and DSC thermograms (**B**,**D**,**F**) of GAL with PEO, HPMC, and EC (F1–F3). FTIR revealed band shifts suggesting interactions, while DSC showed altered thermal transitions, indicating changes in crystallinity and compatibility relevant for formulation.

The spectroscopic (FTIR) and thermal (DSC) results for the binary mixtures of GAL hydrobromide with the controlled-release polymers (PEO, HPMC, and EC) are presented in Figure 3.

FTIR spectra (Figure 3A,C,E) and DSC thermograms (Figure 3B,D,F) of binary mixtures of GAL with three polymer excipients—PEO, HPMC and EC—illustrate potential interactions and thermal behavior changes. FTIR profiles reveal shifts or overlaps in characteristic absorption bands in the binary formulations (F1: GAL + PEO; F2: GAL + HPMC; F3: GAL + EC), suggesting possible hydrogen bonding or physical interactions. DSC analysis shows altered thermal transitions in binary mixtures compared to individual components, with modifications in melting points or disappearance of endothermic peaks, indicating changes in crystallinity or compatibility between GAL and each polymer. These results support the use of these excipients in solid dosage formulation development.

In the case of the mixture composed of GAL hydrobromide and polymer PEO (F1), the FTIR spectrum (Figure 3A) exhibited characteristic bands of both components, including the C–H stretching between 2800 and 3000 cm^−1^ and the asymmetric C–O–C stretching between 1100 and 1150 cm^−1^. There was a noticeable attenuation in the intensity of the N–H band (~3520 cm^−1^) of the GAL ammonium cation, as well as in the ether group of polymer PEO, suggesting the presence of weak hydrogen bond-type interactions between both components [75]. Consistently, its DSC thermogram (Figure 3B) showed persistence of the PEO melting endotherm signal at 68.5 °C, although with a slight reduction in intensity and a shift to 66 °C. Notably, the GAL hydrobromide melting signal at 260 °C and the signal at 270 °C were no longer observed, replaced by a signal at a broad endothermic event at 220–230 °C and an exothermic event at 265–275 °C. This is indicative of drug dissolution or amorphization within the polymer matrix upon heating, an effect similarly observed in DSC studies where the API peak vanishes in solid dispersions or intimate mixtures with PEG/PEO [76,77,78,79] and EC matrix tablets [80].

In the case of the F2 mixture (GAL hydrobromide + polymer HPMC), the FTIR spectrum (Figure 3C) retained the main bands of both components but showed a significant decrease in the intensity of the N–H stretching (~3520 cm^−1^), along with a broadening of the O–H band (3300–3500 cm^−1^) of HPMC. These changes may be associated with hydrogen bond-type interactions between the hydroxyl groups of the polymer and the ammonium group of GAL, in accordance with previous studies on HPMC-based hydrogel [81]. Furthermore, the DSC thermogram (Figure 3D) evidenced the disappearance of the GAL melting signal (260 °C) related to the effect previously mentioned, while the glass transition of polymer HPMC (185–195 °C) shifted slightly to approximately 180 °C. These alterations could be related to disruption in the structural organization of the polymer and the drug, resulting from molecular-level interactions that promote the formation of an amorphous solid dispersion [82].

Finally, the F3 mixture (GAL hydrobromide + polymer EC) exhibited a distinct behavior. The FTIR spectrum (Figure 3E) preserved the bands attributed to C–H (2900–3000 cm^−1^), C–O–C (1150 cm^−1^) of polymer EC, and N–H of GAL hydrobromide (~3520 cm^−1^) without notable shifts, indicating the absence of strong molecular interactions between the components [83]. Consequently, the DSC thermogram (Figure 3F) showed persistence of both the EC (172–178 °C) and the GAL melting signals, though with slight broadening and reduced intensity. This thermal stability suggests that F3 behaves as a physically dispersed system, with no evidence of thermal interference or molecular complexation, as described in inert polymer EC–drug formulations [84]. In this manner, the binary mixture of HPMC and GAL exhibited pronounced spectral and thermal changes, including shifts in IR bands associated with functional groups (N–H stretching of GAL and the O–H band of HPMC), as well as alterations in the GAL melting signal and the glass transition temperature of the HPMC polymer. These findings suggest the formation of molecular-level interactions, most likely hydrogen bonding between the polymer and the drug and drug dissolution or amorphization within the polymer matrix. In contrast, the PEO–GAL combination showed more moderate modifications, such as attenuation of the N–H band intensity in the IR spectrum, indicating a weaker or partial interaction. Meanwhile, the EC–GAL mixture exhibited no significant spectral changes, suggesting that this polymer behaves as an inert excipient, without establishing meaningful interactions with GAL. Taken together, these results suggest that the interaction strength between GAL and the polymers follows the order HPMC > PEO > EC.

### 3.2. Physical Analysis of Powder Excipients and Formulations

#### 3.2.1. Particle Size and Size Distribution

The particle size and size distribution (Span) data for the individual excipients and formulation blends are summarized in Table 2. The results showed notable differences regarding the materials evaluated. The active pharmaceutical ingredient, GAL hydrobromide, displayed the highest particle size variability, with a large Dv90 (654.0 µm) (Figure S1) and a pronounced polydispersity (Span = 3.5). These values are consistent with the broad particle size distributions reported for crystalline alkaloid salts in solid-state formulations [85]. In contrast, PEO exhibited a monodisperse profile (Span = 1.0) despite its large average particle size (Dv50 = 634.7 µm) (Figure S2), suggesting greater uniformity in granule distribution. Similar particle size behavior has been reported for high molecular weight PEOs used in matrix [86].

For the remaining controlled-release polymers, HPMC and EC showed moderately polydisperse profiles, with Span values of 1.7 and 1.8, respectively. These were associated with intermediate particle sizes for HPMC (Dv50 = 263.7 µm) (Figure S3) and markedly smaller particles in the case of EC (Dv50 = 6.2 µm) (Figure S4). The particle size distribution of HPMC agrees with those values reported by Jeong et al. (2019) for agglomerated polymeric systems [87], while the particle size of polymer EC is consistent with that reported for fine particle Ethyl cellulose [88]. Regarding the diluent excipients, Starch^®^ 1500 and spray-dried lactose monohydrate, these exhibited broader distributions (Span = 2.9 and 2.2, respectively) (Figures S5 and S6), indicating higher particle size heterogeneity. These values are comparable to those reported for partially pregelatinized starches and spray-dried lactose in pharmaceutical formulations [89,90].

Regarding the formulation blends, particle size and polydispersity varied substantially depending on the polymer used. Formulation 1 (GAL + PEO + excipients) displayed the highest heterogeneity (Span = 3.7) and a broad particle size interval (Dv10 = 13.9 µm; Dv90 = 316.4 µm) (Figure S7), which may suggest the presence of both fine and coarse particles due to component mismatch. On the other hand, Formulation 2 (GAL + HPMC + excipients) exhibited a more uniform size distribution (Span = 2.3) and intermediate particle size (Dv50 = 146.2 µm) (Figure S8), while Formulation 3 (GAL + EC + excipients) showed the smallest mean particle size (Dv50 = 79.7 µm) (Figure S9) combined with moderate polydispersity (Span = 2.9).

These findings confirm that the particle size distribution of the final blends is not solely determined by the polymeric component, but is significantly influenced by interactions with other excipients and the resulting granule architecture formed during blending. While each polymer exhibits a characteristic particle size and polydispersity profile—such as the large, monodisperse granules of PEO or the fine, narrowly distributed particles of EC—the incorporation of additional excipients, including diluents like Starch^®^ 1500 and spray-dried lactose, alters the overall granulometric behavior of the mixture [91]. For example, Formulation 1 (GAL + PEO + excipients) displayed a high degree of heterogeneity (Span = 3.7) despite PEO’s inherently narrow distribution (Span = 1.0), suggesting that mismatches in particle size and morphology—such as combining coarse polymer granules with finer excipients—can lead to broader distributions and irregular packing [92]. In contrast, Formulation 2 (GAL + HPMC + excipients) exhibited a more balanced size distribution (Span = 2.3), likely due to better size compatibility between polymer and excipients, resulting in a more cohesive and uniform blend. Similarly, Formulation 3 (GAL + EC + excipients), despite using the finest polymer (EC, Dv50 = 6.2 µm), showed intermediate heterogeneity (Span = 2.9), indicating that particle size compatibility—as well as shape, density, and surface characteristics—also governs blending behavior. Therefore, the final granulometric profile of the formulation cannot be attributed solely to the polymer, but arises from complex physical interactions among all components, collectively defining the blend’s structure, flow, and compressibility [93].

#### 3.2.2. Granulometric Characterization Based on Flowability Indices and Porosity

The results of the granulometric characterization based on flowability Indices (Carr index-CI and Hausner ratio-HR), as well as porosity (ε%), are summarized in Table 3.

The Carr index (CI), Hausner ratio (HR), and porosity (ε%) are fundamental parameters for evaluating the granulometric behavior of powder materials, particularly in terms of their flowability and interparticle cohesion. According to USP (2023), CI values below 15% indicate excellent flow, values between 15 and 25% suggest moderate flow, and values above 25% reflect high cohesiveness and poor flow. Similarly, HR values close to 1.00 are associated with good flow, values between 1.25 and 1.40 with fair flow, and values above 1.40 are linked to agglomeration tendencies. Porosity, in turn, describes the volume of voids within the powder bed and affects packing efficiency and air retention, which can hinder mixing and the uniform flow of the system [94].

Regarding the diluent excipients employed in the formulation, spray-dried lactose monohydrate exhibited the most favorable flow behavior (CI = 3%, HR = 1.03), consistent with excellent flowability and moderate porosity (56.63%). These values agree with those reported by Shah et al. (2008) in binary mixtures [85]. In contrast, Starch^®^ 1500 showed moderate flow (CI = 21%, HR = 1.26) and low porosity (50.94%), suggesting higher bulk density and reduced air entrapment. These findings agree with those of Rojas et al. (2012) for partially pregelatinized starches [95].

Among the controlled-release polymers, different flow behaviors were observed. In the case of polymer PEO, it showed good flow (CI = 12%, HR = 1.14) and high porosity (62.27%), which may favor dispersion during blending but with lower packing efficiency. These values are comparable to those reported by Castañeda Hernández (2023) for high molecular weight PEO [96]. HPMC exhibited the highest cohesion (CI = 21%, HR = 1.26), as well as the highest porosity (78.41%) among all formulation ingredients evaluated in this study, suggesting poor packing efficiency and high air retention. This trend is consistent with the observations of Jeong et al. (2019), who reported high porosity and reduced flow in systems containing HPMC [87]. EC showed intermediate behavior, with moderate cohesion (CI = 15%, HR = 1.18) and high porosity (73.16%), which could lead to irregular restructuring under compression. This behavior is like that described by Kar et al. (2025) in matrix systems containing polymer EC [97].

Concerning the blend formulations, these showed notable differences due to the interaction between the controlled-release polymers (PEO, HPMC, and EC) and the rest of the formulation ingredients (GAL hydrobromide and excipients). In the case of Formulation 1 (GAL + PEO + excipients), it exhibited the poorest flow profile (CI = 46%, HR = 1.87) and high porosity (70.86%), indicating elevated cohesion and poor packing efficiency, possibly due to particle size differences and deformability. In contrast, Formulation 2 (GAL + HPMC + excipients) presented the best flow behavior (CI = 12%, HR = 1.13) and the lowest porosity (55.15%), suggesting lower internal friction and better granular rearrangement, despite the high porosity of pure HPMC. Formulation 3 (GAL + EC + excipients) showed intermediate behavior (CI = 29%, HR = 1.41) with high porosity (67.17%), describing a system with limited flow and open packing. Each of these findings reinforces that polymer composition and excipient compatibility significantly influence the bulk behavior of powder systems. Besides, the values obtained are consistent with previous studies on similar pharmaceutical formulations, supporting the consistency and robustness of the experimental analysis.

#### 3.2.3. Compressibility, Compactability, and Tabletability Analysis

**Figure 4:**
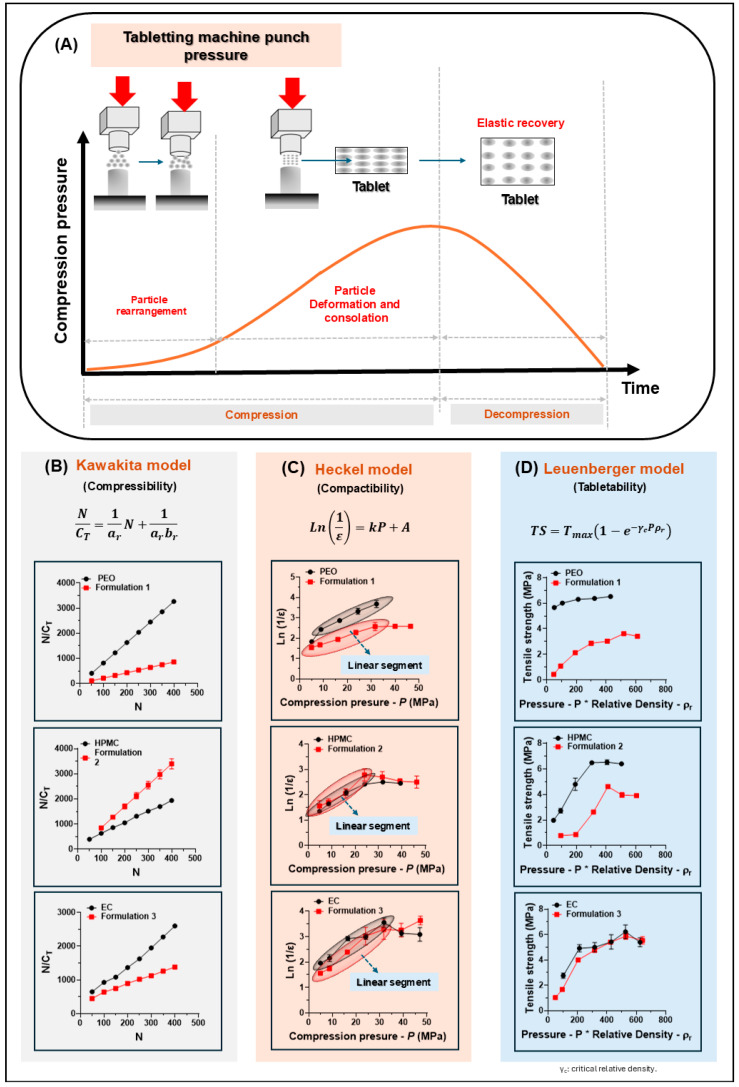
Compressional modeling of GAL formulations with PEO (F1), HPMC (F2), and EC (F3). (**A**) shows powder compaction stages. (**B**) Kawakita model assessed compressibility, (**C**) Heckel model compactability, and (**D**) Leuenberger model tabletability, highlighting polymer effects on tablet strength and compression behavior.

The results of the compressibility, compactibility, and tabletability analysis carried out through the Kawakita, Heckel, and Leuenberger models are shown in Figure 4, while the different parameters obtained from these models are summarized in Table 4. In relation to Figure 4A, it shows a schematic adapted from the article by Ren et al. (2024) [98], which illustrates the three fundamental stages of pharmaceutical powder compaction: This conceptual framework allows associating the three classical mechanical models (Kawakita, Heckel, and Leuenberger) with different phases of the compaction phenomenon. In this context, a first stage corresponds to the low-pressure range (typically between 1 and 20 MPa), where the Kawakita model (Figure 4B) describes powder densification behavior through the intercept-derived $a_{T}$ parameter (also referred to as the compressibility constant or volume reduction capacity), with values above 0.5 indicating good compressibility and those below 0.3 suggesting poor densification ability [99]. The slope-derived $b_{T}$ parameter (also known as the rearrangement resistance factor or pressure term) reflects how easily particles rearrange under stress; lower values (0.01–0.02 MPa^−1^) indicate free particle movement, while higher values (>0.04 MPa^−1^) suggest hindered rearrangement. The $P_{k}$ calculated as 1/$b_{T}$, denotes the pressure needed to reduce bed volume by 50%; values under 50 MPa imply efficient compaction onset, while pressures above 100 MPa reflect structural rigidity.

In the intermediate pressure range (20–50 MPa), the Heckel model (Figure 4C) assesses plastic deformation based on the linearized relation ln(1/ε) vs. pressure. From this, the *A* parameter (intercept) describes the initial packing efficiency or extent of rearrangement prior to plastic deformation, with values above 1.0 often linked to loosely packed beds and those near 0.0 indicating efficient initial packing. The *K* parameter (slope), also known as the yield pressure coefficient, reflects the rate of plastic densification. Its reciprocal, $P_{y}$, represents the mean yield pressure at which particles begin to deform plastically. In general, lower $P_{y}$ values (typically below 80 MPa) denote high plasticity and ease of deformation (e.g., microcrystalline cellulose), while higher $P_{y}$ values (commonly above 120 MPa) are associated with brittle behavior and poor plastic flow [100].

Figure 4A illustrates the powder compaction process in a tableting machine, progressing through particle rearrangement, deformation, and densification under pressure, followed by elastic recovery during decompression. The mechanical behavior of three galantamine-based formulations containing different polymers—PEO (Formulation 1), HPMC (Formulation 2), and EC (Formulation 3)—was analyzed using three well-established compaction models. The Kawakita model (Figure 4B) evaluates compressibility by plotting the relationship between applied pressure (N) and volume reduction (N/C), showing linear trends for all formulations. The Heckel model (Figure 4C) assesses compactability, with linear segments indicating plastic deformation under increasing compression pressure. The Leuenberger model (Figure 4D) examines tabletability, relating tensile strength to pressure-relative density, highlighting the influence of each polymer on the resulting tablet strength. Together, these models provide quantitative insights into powder behavior during compression and support rational formulation development.

In relation to the Leuenberger model (Figure 4D) quantifies tabletability via an exponential saturation curve, with $T_{max}$ (intercept or maximum tensile strength) defining the upper mechanical limit of the compacted system. Systems with $T_{max}$ > 3 MPa are generally considered mechanically robust, while those below 1.5 MPa may present fragile structures. The $\gamma_{c}$ parameter (slope), or critical relative density, identifies the density at which 50% of $T_{max}$ is attained; efficient systems reach this threshold at $\gamma_{c}\;$≈ 0.85, while values above 0.93 may suggest delayed or inefficient strength buildup [101]. The trends observed across all models were consistent with their mechanistic assumptions: Kawakita plots demonstrated linear N/C vs. N relationships, indicative of uniform compressibility; Heckel profiles revealed defined linear regions within 20–50 MPa, denoting plastic deformation domains; and Leuenberger curves displayed characteristic exponential growth in tensile strength with rising pressure.

Regarding system-specific behavior, polymer PEO exhibited moderate compressibility and plasticity but limited tensile strength, likely due to its lubricity reducing interparticle bonding efficiency. Polymer HPMC showed similar responses between its pure and formulated forms, reflecting minimal structural alteration with excipient addition. Polymer EC displayed low compressibility and strength in its pure state, but improved dramatically when formulated with GAL and excipients. Among the Formulations, systems 1 and 2 demonstrated balanced compaction behavior, while Formulation 3 achieved the highest values in all models, particularly in $T_{max}$, confirming its superior mechanical integrity.

The results obtained from each of the three models studied allow for a multidimensional and holistic understanding of powder mechanics. In this regard, the values for each of the parameters of the Kawakita, Heckel, and Leuenberger models are summarized in Table 4. According to the parameter values obtained from the Kawakita model, the polymer PEO exhibited an intercept $a_{T}$ of 0.14, which indicates limited compressibility associated with its semi-rigid nature and low interparticle cohesion; whereas, when mixed with GAL hydrobromide and the remaining excipients (Formulation 1), this value markedly increased to 0.47, suggesting a significant improvement in packing capacity, most likely facilitated by API redistribution and its lubricating effect. Similarly, the slope $b_{T}$ increased from 0.28 MPa^−1^ to 1.20 MPa^−1^, indicating a higher degree of particle rearrangement; consequently, the Kawakita pressure ($P_{k}$) decreased from 4.21 MPa to 1.00 MPa, which is consistent with highly deformable systems such as microcrystalline cellulose [99]. In contrast, the polymer HPMC showed an intercept $a_{T}$ of 0.23 in its pure state, which was reduced to 0.13 when mixed with the formulation components (Formulation 2). This result may be explained by an increase in structural cohesiveness, likely mediated by hydrophilic interactions. Similarly, its slope $b_{T}$ increased slightly (0.02 to 0.04 MPa^−1^), while $P_{k}$ dropped from 43.46 to 26.86 MPa, suggesting more efficient compaction due to morphological adjustments. In the case of the polymer EC, it exhibited an opposite behavior, where its intercept $a_{T}$ increased from 0.18 to 0.39 when mixed with the remaining formulation components (Formulation 3). This result, which could initially suggest favorable compressibility, is contradicted by the slope $b_{T}$, which decreased from 0.02 to 0.01 MPa^−1^, leading to a sharp rise in $P_{k}$ (56.78 to 138.69 MPa). This result suggests a significant resistance to initial packing, attributable to the polymer’s hydrophobic nature and lack of molecular affinity [54,102].

Regarding the Heckel model (linear segment), the intercept values *A*, which are associated with initial packing density or pre-deformation porosity, showed notable differences. Polymers PEO (*A* = 1.95) and EC (*A* = 1.69) exhibited higher initial porosity, which decreased to *A* = 1.33 and *A* = 1.21, respectively, when blended with the rest of the formulation ingredients, suggesting improved reorganization into the powder particle system. In contrast, polymer HPMC presented a denser initial packing (A = 1.10), which remained relatively unchanged in the formulation (*A* = 1.16). In terms of the slope *K*, which relates to plastic deformation capacity, polymer PEO showed a reduction from 0.055 to 0.039 MPa^−1^, leading to an increase in yield pressure ($P_{y}$ = 25.85 MPa). Conversely, polymers HPMC and EC showed increased *K* values upon formulation (HPMC: 0.056 to 0.067 MPa^−1^; EC: 0.058 to 0.071 MPa^−1^), resulting in lower yield pressures ($P_{y}$ = 14.91 and 14.03 MPa, respectively). These findings suggest enhanced plasticity induced by the formulation components, potentially due to multiple hydrophilic interactions (in HPMC) or improved physical redistribution (in EC).

Concerning the Leuenberger model, tabletability differences were also evident between the individual polymers and their respective formulations. Pure PEO showed high mechanical strength ($T_{max}$ = 6.33 MPa), which decreased to 4.00 MPa after formulation, although it exhibited earlier mechanical consolidation ($\gamma_{c}$ = 0.0037). Similarly, HPMC maintained robust mechanical performance ($T_{max}$ = 7.01 MPa pure; 4.61 MPa in Formulation 2), along with improved structural efficiency as exhibited by a lower $\gamma_{c}$. In contrast, polymer EC demonstrated a remarkable shift in its pure form; it exhibited poor tabletability ($T_{max}$ = 0.95 MPa); but when it was combined with the rest of the formulation ingredients (Formulation 3), it reached 6.30 MPa, achieving values comparable to modified robust matrices [103]. Consistently, all formulations exhibited low $\gamma_{c}$ values (~0.0037–0.0045), describing an efficient structural consolidation at low relative densities.

All these results demonstrate that the mechanical behavior of the pure polymers is primarily governed by their intrinsic structure, but once they are mixed with the rest of the formulation ingredients, the compaction properties can be significantly modulated. In this context, based on the models evaluated, the system with the best mechanical performance corresponds to Formulation 3 (Gal + EC + excipients), followed by Formulation 2 (Gal + HPMC + excipients), then Formulation 1 (Gal + PEO + excipients), continuing with the pure polymers in the order HPMC, PEO and EC, which was the least favorable in its individual state.

### 3.3. Galantamine Modified-Release Tablets Characterization

#### 3.3.1. Tablet Physical Characterization

The physical characteristics of GAL modified-release tablets formulated with the polymers PEO, HPMC, and EC are presented in Table 5. In relation to average weight, Formulations 1 (GAL + PEO + excipients) and 2 (GAL + HPMC + excipients) exhibited comparable values (200.62 ± 1.85 mg and 201.27 ± 2.72 mg, respectively), indicating good content uniformity during compaction. Formulation 3 (GAL + EC + excipients) showed a significantly lower mean weight (176.03 ± 4.50 mg), likely due to higher initial porosity, as identified in the previous Heckel analysis. Regarding mechanical resistance, Formulation 3 (GAL + EC + excipients) showed the highest hardness (0.1143 ± 0.0043 kN), closely followed by Formulation 2 (GAL + HPMC + excipients) with 0.1058 ± 0.0028 kN, which fit with the predictions of the Leuenberger model, suggesting efficient early consolidation. In contrast, Formulation 1 (GAL + PEO + excipients) exhibited lower hardness (0.0576 ± 0.0035 kN), which is consistent with its limited plastic deformation capacity. All formulations complied with pharmacopeial friability limits [104]. Formulation 1 (GAL + PEO + excipients) exceeded 4 h, describing a controlled erosion mechanism; although Formulation 3 (GAL + EC + excipients) exhibited the highest hardness, its matrices disintegrated within only 30 min, indicating a porous internal structure and limited matrix cohesion, as previously described [105]. This behavior can be explained by the fact that tablet hardness, measured under dry conditions, primarily reflects the mechanical resistance to fracture during handling and does not necessarily predict the structural integrity of the hydrated matrix [104]. EC, being a water-insoluble and non-swelling polymer, fails to form a cohesive gel barrier, thereby facilitating the dissolution and leaching of soluble excipients and drug particles [29,106]. This process increases porosity and weakens the compact structure, allowing rapid penetration of the dissolution medium and promoting matrix erosion or fragmentation, which accounts for the poor release control observed.

#### 3.3.2. In Vitro Dissolution Tests

The percentages of GAL released at different times and their compliance with USP acceptance criteria are shown in Table 6.

Regarding compliance, Formulations 1 (GAL + PEO + excipients) and 2 (GAL + HPMC + excipients) conformed to the USP specifications at 1, 4, and 12 h, showing sustained and modulated release behavior. These findings agree with matrix systems based on swelling and gel formation dynamics. In contrast, Formulation 3 (GAL + EC + excipients) exceeded the USP limits at both 1 and 4 h, releasing more than 76 % of GAL in each case. Although it fulfilled the 12-h requirement (78.2 %), this premature release pattern indicates a lack of diffusional control and matrix integrity. In relation to the physicochemical properties of polymer EC, these results support the hypothesis of low polymer–API affinity and poor cohesive behavior, as reported by Verma et al. [107].

**Figure 5:**
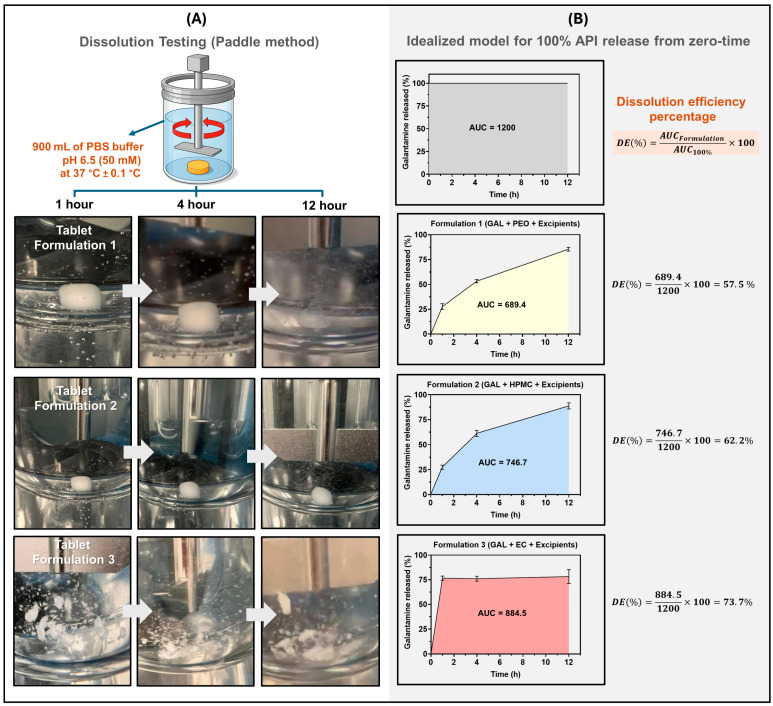
Cumulative release of GAL from tablets over 12 h. (**A**) shows dissolution testing with images at 1, 4, and 12 h. (**B**) release profiles and dissolution efficiencies (DE%): F1 (GAL + PEO) = 57.5%, F2 (GAL + HPMC) = 62.2%, F3 (GAL + EC) = 73.7%, highlighting polymer effects on release performance.

In relation to the cumulative release profiles of GAL and the dissolution efficiency (DE%) values for each formulation are presented in Figure 5.

In vitro dissolution testing of three GAL tablet formulations (Figure 5A) was conducted using the paddle method in 900 mL of phosphate-buffered saline (PBS, pH 6.5, 50 mM) at 37 °C ± 0.1 °C. Photographs were taken at 1, 4, and 12 h to document the disintegration behavior of each formulation over time. Dissolution profiles (Figure 5B) compare the percentage of GAL released over 12 h against an idealized model representing 100% release from time zero (area under the curve, AUC = 1200). Dissolution efficiency (DE%) was calculated for each formulation: Formulation 1 (GAL + PEO) showed a DE of 57.5%, Formulation 2 (GAL + HPMC) achieved 62.2%, and Formulation 3 (GAL + EC) reached the highest efficiency at 73.7%. These results demonstrate the impact of polymer excipients on GAL release kinetics and overall dissolution performance.

The values obtained of dissolution efficiency-DE% were 57.5 % for Formulation 1 (GAL + PEO + excipients), 62.2 % for Formulation 2 (GAL + HPMC + excipients), and 73.7 % for Formulation 3 (GAL + EC + excipients). In relation to formulation performance, only systems based on polymers PEO and HPMC maintained a coherent association between release profiles and physical behavior. These results are consistent with the release mechanism proposed by Yarce et al. (2016), where dissolution efficiency is linked to molecular interaction, surface energy, and structural cohesion within the matrix [65]. In the case of Formulation 2 (GAL + HPMC + excipients), this demonstrated the most robust performance, combining structural integrity, non-disintegration, and a modulated release profile, while Formulation 1 (GAL + PEO + excipients) showed slightly lower efficiency but maintained gradual erosion and sustained drug delivery. Conversely, Formulation 3 (GAL + EC + excipients) exhibited a high DE%; however, this result was primarily associated with an initial burst release and insufficient matrix cohesion, which led to a release profile that deviated from the intended modified-release performance. This behavior has been previously described as a limitation in matrix systems, where poor polymer–API interaction and structural weakness result in premature release [108]. Additionally, some researchers have indicated that the accelerated and uncontrolled release of the API from matrices containing EC is associated with polymer volume fractions below its percolation threshold. In this subcritical regime, the hydrophobic phase fails to form a continuous three-dimensional network, thereby reducing the tortuosity of the diffusion pathways and facilitating unrestricted drug transport [109]. Grund et al. (2014) reported a percolation threshold of 65% *v*/*v* for EC [31], a value higher than the EC proportion employed in the present study.

Similarly, studies conducted on EC-based matrix tablets, using concentrations and viscosity grades comparable to those evaluated herein, have documented a rapid release, with approximately 90% of the API released within only 1.0 to 2.5 h [110]. In contrast, literature reports indicate that controlled release from EC matrices is generally achieved with polymer proportions exceeding 50% *w*/*w* [80]. In summary, although Formulation 3 showed the highest dissolution efficiency, its release behavior did not correspond to a sustained profile due to deficient structural integrity and rapid API diffusion. In contrast, Formulations 1 and 2 achieved a functional balance between physical robustness and controlled release, which is consistent with other similar studies carried out [65]. These findings confirm that DE% must be interpreted in relation to the intended release profile and not as an isolated performance metric.

Furthermore, the compliance of HPMC- and PEO-based formulations with USP dissolution criteria demonstrates significant clinical relevance, as it guarantees the reproducibility and reliability of extended-release performance throughout the intended dosing interval [111]. This pharmacokinetic consistency is critical for maintaining stable plasma concentrations of galantamine, thereby optimizing therapeutic outcomes, minimizing concentration-dependent adverse effects, and promoting patient adherence, particularly important in the long-term management of neurodegenerative disorders such as Alzheimer’s disease [112].

## 4. Conclusions

This study demonstrated a clear relationship between the physicochemical, granulometric, and functional performance properties of three widely used polymers—PEO, HPMC and EC—when employed as modified-release matrices in solid oral dosage forms containing GAL. Spectroscopic (FTIR) and thermal (DSC) analyses revealed the presence of physical interactions between GAL and the polymeric matrices, without evidence of critical incompatibilities or degradation processes. Although these interactions were not disruptive, these interactions were directly associated with the drug release mechanism and the cohesive structure of the system.

Mechanically, HPMC provided the highest compactability and compressive strength, forming robust, non-disintegrating tablets with modulated drug release. Flow-related parameters (Carr Index, Hausner Ratio, porosity-ε) and compressibility models (Heckel, Kawakita, and Leuenberger) confirmed that HPMC-based combined low interparticle cohesion with high plastic deformation capacity, favoring the formation of dense and stable matrices.

PEO showed intermediate mechanical performance, with suitable plastic deformation, high initial densification (Kawakita model), and sustained drug release via surface erosion. In contrast, EC showed high powder cohesiveness and tablet hardness, but poor particle–matrix interaction and low plasticity (based on Heckel and Leuenberger analyses), resulting in low internal cohesion, early disintegration, and uncontrolled GAL release.

According to pharmaceutical performance, only the formulations based on the polymers HPMC and PEO complied with USP criteria and fulfilled the objectives of modified release. Despite showing higher dissolution efficiency (DE%) values, the formulations based on the polymer EC failed to adequately control the drug release profile. Overall, these findings demonstrate that integrating spectroscopic and thermal characterization, granulometric data, compressibility models, and functional performance are essential for the rational and robust design of polymer-based oral matrices for controlled GAL delivery. Future work should explore the scalability of these formulations and assess their in vivo performance to support clinical translation of optimized GAL delivery systems.

## Figures and Tables

**Table 1:** Composition of GAL hydrobromide extended-release tablets.

Formulation Ingredient	Formulation Function	% *w*/*w*		
		Tablet Formulation 1	Tablet Formulation 2	Tablet Formulation 3
Galantamine HBr (GAL)	API	4.44	4.44	4.44
Polyethylene oxide (PEO)	Controlled-release polymer	31.52	--	--
Hydroxypropyl methylcellulose (HPMC)	Controlled-release polymer	--	31.52	--
Ethyl cellulose (EC)	Controlled-release polymer	--	--	31.52
Starch^®^ 1500	Diluent 1	31.52	31.52	31.52
Spray-dried lactose monohydrate	Diluent 2	31.52	31.52	31.52
Colloidal silicon dioxide	Lubricant	0.5	0.5	0.5
Magnesium stearate	Glidant	0.5	0.5	0.5

API: Active pharmacological ingredient.

**Table 2:** Particle size distribution parameters (Dv10, Dv50, Dv90, and Span) for individual ingredients and their corresponding formulation blends (mean ± SD).

Formulation Ingredients and Tablet Formulations	Dv10 (µm)	Dv50 (µm)	Dv90 (µm)	Span
GAL	74.4	166.9	654.0	3.5
Polyethylene oxide (PEO)	375.1 ± 16.2	634.7 ± 5.4	1028.3 ± 39.3	1.0 ± 0.1
Hydroxypropyl methylcellulose (HPMC)	65.4 ± 4.4	263.7 ± 4.2	516.5 ± 11.0	1.7 ± 0.1
Ethyl cellulose ether (EC)	1.9 ± 0.0	6.2 ± 0.1	13.0 ± 0.4	1.8 ± 0.0
Starch^®^ 1500	14.8 ± 0.2	73.1 ± 2.8	229.6 ± 7.2	2.9 ± 0.0
Spray-dried lactose monohydrate	40.8 ± 8.0	110.9 ± 14.1	282.3 ± 73.6	2.2 ± 0.3
Formulation 1	13.9 ± 0.7	81.5 ± 10.6	316.4 ± 54.8	3.7 ± 0.2
Formulation 2	27.1 ± 5.9	146.2 ± 29.3	358.6 ± 27.8	2.3 ± 0.3
Formulation 3	8.6 ± 0.3	79.7 ± 3.2	241.0 ± 8.1	2.9 ± 0.0

**Table 3:** Flowability indices (Carr Index, Hausner Ratio) and porosity (ε%) of formulation ingredients and final blends (mean ± SD).

Formulation Ingredients and Tablet Formulations	Carr Index (%)	Hausner Ratio	Porosity-ε (%)
Polyethylene oxide (PEO)	12 ± 0.25	1.14 ± 0.00	62.27 ± 0.70
Hydroxypropyl methylcellulose (HPMC)	21 ± 0.33	1.26 ± 0.01	78.41 ± 0.34
Ethyl cellulose ether (EC)	15 ± 0.24	1.18 ± 0.00	73.16 ± 0.41
Starch^®^ 1500	21 ± 0.66	1.26 ± 0.01	50.94 ± 1.56
Spray-dried lactose monohydrate	3 ± 0.10	1.03 ± 0.00	56.63 ± 1.40
Formulation 1 (GAL + PEO + Excipients)	46 ± 1.66	1.87 ± 0.06	70.86 ± 1.04
Formulation 2 (GAL + HPMC + Excipients)	12 ± 0.69	1.13 ± 0.01	55.15 ± 2.64
Formulation 3 (GAL + EC + Excipients)	29 ± 0.64	1.41 ± 0.01	67.17 ± 0.73

**Table 4:** Kawakita, Heckel, and Leuenberger parameters of the evaluated ingredients and formulations.

**Ingredients and** **Formulations**	**Parameters of the Kawakita Linear Function**			
	$\frac{N}{C_{T}}=\frac{N}{a_{T}}+\frac{1}{a_{T}b_{T}},(N:Applied\;pressure,\;C_{T}:\;Degree\;of\;volume\;reduction)$			
	$a_{T}$	$b_{T}$	Pk=1bT	**R^2^**
	(Obtained from Intercept)	(Obtained from Slop)		
	Compressibility constant	Pressure term	Kawakita pressure	
PEO	0.14 ± 0.00	0.28 ± 0.15	4.21 ± 2.02	1.0000
HPMC	0.23 ± 0.00	0.02 ± 0.00	43.46 ± 2.80	0.9995
EC	0.18 ± 0.01	0.02 ± 0.00	56.78 ± 7.10	0.9961
Formulation 1	0.47 ± 0.00	1.20 ± 0.66	1.00 ± 0.49	0.9999
Formulation 2	0.13 ± 0.01	0.04 ± 0.02	26.86 ± 11.86	0.9837
Formulation 3	0.39 ± 0.01	0.01 ± 0.00	138.69 ± 3.11	0.9955
**Ingredients and** **formulations**	**Parameters of the Heckel linear function**			
	$\mathrm{Ln}\left(\frac{1}{1-D}\right)=K*P+A,\;(D:\;Relative\;density,\;P:\;Applied\;pressure)$			
	***A***	***K***	Py=1K	**R^2^**
	(Intercept)	(Slop)		
	Initial packing behavior	Compressibility coefficient	Yield Pressure	
PEO	1.94788 ± 0.03269	0.05500 ± 0.0018	18.195 ± 0.486	0.99786
HPMC	1.09569 ± 0.04689	0.05589 ± 0.00342	17.937 ± 0.898	0.99255
EC	1.69532 ± 0.07564	0.05754 ± 0.00434	17.446 ± 1.077	0.98324
Formulation 1	1.3325 ± 0.02682	0.03874 ± 0.00167	25.845 ± 0.911	0.99446
Formulation 2	1.15816 ± 0.06378	0.06726 ± 0.00473	14.917 ± 0.859	0.99022
Formulation 3	1.21027 ± 0.02513	0.07138 ± 0.00318	14.028 ± 0.511	0.99407
**Ingredients and** **formulations**	**Leuenberger parameters**			
	$TS=T_{max}*(1-e^{\left(-\gamma_{c}*P*\rho_{r}\right)}),\;(TS:\;Tensil\;strength,\;P:\;Compaction\;pressure,\;\rho_{r}:\;Relative\;density)\;$			
	$T_{max}$	$\gamma_{c}$		**R^2^**
	(Intercept)	(Slop)		
	Maximum Tensile Strength	Critical Relative Density		
PEO	6.33247 ± 0.09791	0.04141 ± 0.0055		1.0000
HPMC	7.01417 ± 0.41633	0.00622 ± 0.00102		1.0000
EC	0.94655 ± 0.0111	0.04205 ± 0.00527		1.0000
Formulation 1	4.00407 ± 0.31389	0.00373 ± 6.59635 × 10^−4^		1.0000
Formulation 2	4.60899 ± 0.7821	0.00455 ± 0.00199		0.9999
Formulation 3	6.2971 ± 0.41643	0.00432 ± 7.27968 × 10^−4^		1.0000

PEO: Polyethylene oxide, HPMC: Hydroxypropyl methylcellulose, EC: Ethyl cellulose ether.

**Table 5:** Physical characterization of GAL modified-release tablets formulations.

Tablet Formulation	Weight (mg)	Hardness (kN)	Friability (%)	Disintegration Time
*p*-Value	<0.0001	<0.0001	0.072	-
Formulation 1	200.62 ± 1.85	0.0576 ± 0.0035	0.1 ± 0.1	>4 h
Formulation 2	201.27 ± 2.72	0.1058 ± 0.0028	0.4 ± 0.3	Does not happen
Formulation 3	176.03 ± 4.50	0.1143 ± 0.0043	0.1 ± 0.0	30 min

Formulation 1: (GAL + PEO + Excipients), Formulation 2: (GAL + HPMC + Excipients), Formulation 3: (GAL + EC + Excipients). GAL: galantamine hydrobromide, PEO: Polyethylene oxide, HPMC: Hydroxypropyl methylcellulose, EC: Ethyl cellulose ether.

**Table 6:** Amount of GAL released at different times and acceptance criteria according to the United States Pharmacopeia (USP).

Tablet Formulation	Time (h)	GAL Amount (%)	Meets the Acceptance Criteria
Control	1	20–40	
Formulation 1		27.4	Yes
Formulation 2		27.6	Yes
Formulation 3		76.6	No
Control	4	40–65	
Formulation 1		61.3	Yes
Formulation 2		53.2	Yes
Formulation 3		76.1	No
Control	12	No less than 75	
Formulation 1		88.7	Yes
Formulation 2		85.4	Yes
Formulation 3		78.2	Yes

Formulation 1: (GAL + PEO + Excipients), Formulation 2: (GAL + HPMC + Excipients), Formulation 3: (GAL + EC + Excipients). GAL: galantamine hydrobromide, PEO: Polyethylene oxide, HPMC: Hydroxypropyl methylcellulose, EC: Ethyl cellulose ether.

## Data Availability

All data are available in the manuscript. However, if additional information is required, the corresponding authors may be contacted.

## Supplementary Materials

The following supporting information can be downloaded at: https://www.mdpi.com/article/10.3390/pharmaceutics17091139/s1, Figure S1: Particle size distribution of galantamine hydrobromide (GAL) measured by laser diffraction, shown as volume percentage versus particle size (µm), Figure S2: Particle size distribution of polyethylene oxide (PEO), presented as volume percentage versus particle size (µm), Figure S3: Particle size distribution of hydroxypropyl methylcellulose (HPMC), shown as volume percentage versus particle size (µm), Figure S4: Particle size distribution of ethylcellulose (EC), expressed as volume percentage versus particle size (µm), Figure S5: Particle size distribution of STARCH^®^ 1500, represented as volume percentage versus particle size (µm), Figure S6: Particle size distribution of spray-dried lactose monohydrate, presented as volume percentage versus particle size (µm), Figure S7: Particle size distribution of Formulation 1, showing volume percentage versus particle size (µm), Figure S8: Particle size distribution of Formulation 2, showing volume percentage versus particle size (µm), Figure S9: Particle size distribution of Formulation 3, showing volume percentage versus particle size (µm).

## Abbreviations

The following abbreviations are used in this manuscript:

API	Active Pharmaceutical Ingredient
BCS	Biopharmaceutics Classification System
CI	Carr Index
DE%	Dissolution Efficiency Percentage
DSC	Differential Scanning Calorimetry
EC	Ethylcellulose
FTIR	Fourier Transform Infrared Spectroscopy
GAL	Galantamine
HPLC	High-Performance Liquid Chromatography
HPMC	Hydroxypropyl Methylcellulose
HR	Hausner Ratio
Kp	Kilopond (Tablet Hardness Unit)
PBS	Phosphate Buffered Saline
PEO	Polyethylene Oxide
Py	Yield Pressure
R^2^	Coefficient of Determination (Regression Analysis)
SLS	Static Light Scattering
Tmax	Maximum Tensile Strength
USP	United States Pharmacopeia

## References

[1] Lim A.W.Y., Schneider L., Loy C. (2024). Galantamine for Dementia Due to Alzheimer’s Disease and Mild Cognitive Impairment. Cochrane Database Syst. Rev..

[2] Janssen Research Foundation (2000). Galantamine: Clinical Pharmacology and Biopharmaceutics Review.

[3] Zhao Q., Janssens L., Verhaeghe T., Brashear H.R., Truyen L. (2005). Pharmacokinetics of Extended-Release and Immediate-Release Formulations of Galantamine at Steady State in Healthy Volunteers. Curr. Med. Res. Opin..

[4] Reddi Sree R., Kalyan M., Anand N., Mani S., Gorantla V.R., Sakharkar M.K., Song B.J., Chidambaram S.B. (2025). Newer Therapeutic Approaches in Treating Alzheimer’s Disease: A Comprehensive Review. ACS Omega.

[5] Xing B., Yang J., Hua H., Jiang R. (2025). Rapid Health Technology Assessment of Galantamine for the Treatment of Alzheimer’s Disease: A Review. Medicine.

[6] Jaqua E.E., Tran M.-L.N., Hanna M. (2024). Alzheimer Disease: Treatment of Cognitive and Functional Symptoms. Am. Fam. Physician.

[7] Varadharajan A., Davis A.D., Ghosh A., Jagtap T., Xavier A., Menon A.J., Roy D., Gandhi S., Gregor T. (2023). Guidelines for Pharmacotherapy in Alzheimer’s Disease—A Primer on FDA-Approved Drugs. J. Neurosci. Rural. Pract..

[8] Ono A., Tomono T., Ogihara T., Terada K., Sugano K. (2016). Investigation of Biopharmaceutical and Physicochemical Drug Properties Suitable for Orally Disintegrating Tablets. ADMET DMPK.

[9] Huang F., Fu Y. (2010). A Review of Clinical Pharmacokinetics and Pharmacodynamics of Galantamine, a Reversible Acetylcholinesterase Inhibitor for the Treatment of Alzheimer s Disease, in Healthy Subjects and Patients. Curr. Clin. Pharmacol..

[10] Marucci G., Buccioni M., Ben D.D., Lambertucci C., Volpini R., Amenta F. (2021). Efficacy of Acetylcholinesterase Inhibitors in Alzheimer’s Disease. Neuropharmacology.

[11] Chbib C., Rashid M.A., Shah S.M., Kazi M., Uddin M.N. (2023). Evaluating the Release of Different Commercial Orally Modified Niacin Formulations In Vitro. Polymers.

[12] Wen X., Deng Z., Xu Y., Yan G., Deng X., Wu L., Liang Q., Fang F., Feng X., Yu M. (2021). Preparation and in Vitro/in Vivo Evaluation of Orally Disintegrating/Modified-Release Praziquantel Tablets. Pharmaceutics.

[13] Mubeen I., Zaman M., Farooq M., Mehmood A., Azeez F.K., Rehman W., Akhtar S., Chaudhry M.A., Butt M.H., Shamim Q.U.A. (2022). Formulation of Modified-Release Bilayer Tablets of Atorvastatin and Ezetimibe: An In-Vitro and In-Vivo Analysis. Polymers.

[14] Hollenbeck R.G., Fahmy R., Martinez M.N., Ibrahim A., Hoag S.W. (2025). Design and Process Considerations for Preparation of Modified Release Ivermectin and Praziquantel Tablets by Wet Granulation. AAPS PharmSciTech.

[15] Vlachou M., Siamidi A., Anagnostopoulou D., Christodoulou E., Bikiaris N.N. (2022). Modified Release of the Pineal Hormone Melatonin from Matrix Tablets Containing Poly (L-Lactic Acid) and Its PLA-Co-PEAd and PLA-Co-PBAd Copolymers. Polymers.

[16] Khan B., Choi H.I., Ryu J.S., Noh H.Y., Shah F.A., Khan N., Ansari M.M., Zeb A., Kim J.K. (2024). Core-Shell Tablets Designed for Modified and Sequential Release of Ibuprofen and Rabeprazole. Int. J. Pharm..

[17] Hussain A., Misbah M., Abbas N., Irfan M., Arshad M.S., Shamim R., Bukhari N.I., Mahmood F. (2020). Design and in Vitro Characterization of Orally Disintegrating Modified Release Tablets of Naproxen Sodium. Turk. J. Pharm. Sci..

[18] Kostewicz E.S., Vertzoni M., Benson H.A.E., Roberts M.S. (2021). Oral Drug Delivery for Modified Release Formulations.

[19] Bertocchi P., Antoniella E., Valvo L., Alimonti S., Memoli A. (2005). Diclofenac Sodium Multisource Prolonged Release Tablets—A Comparative Study on the Dissolution Profiles. J. Pharm. Biomed. Anal..

[20] Harrower A.D.B. (1991). Efficacy of Gliclazide in Comparison with Other Sulphonylureas in the Treatment of NIDDM. Diabetes Res. Clin. Pract..

[21] Palmer K.J., Brogden R.N. (1993). Gliclazide: An Update of Its Pharmacological Properties and Therapeutic Efficacy in Non-Insulin-Dependent Diabetes Mellitus. Drugs.

[22] Mandal U., Ray K.K., Gowda V., Ghosh A., Pal T.K. (2010). In-Vitro and in-Vivo Correlation for Two Gliclazide Extended-Release Tablets. J. Pharm. Pharmacol..

[23] Ervasti T., Simonaho S.P., Ketolainen J., Forsberg P., Fransson M., Wikström H., Folestad S., Lakio S., Tajarobi P., Abrahmsén-Alami S. (2015). Continuous Manufacturing of Extended Release Tablets via Powder Mixing and Direct Compression. Int. J. Pharm..

[24] Velasco M.V., Muñoz A., Jiménez-Castellanos M.R., Castellano I., Goñi I., Gurruchaga M. (1996). In Vitro Evaluation of Sustained-Release Matrix Tablets Prepared with New Modified Polymeric Carbohydrates. Int. J. Pharm..

[25] Samy W., Elnoby A., El-Gowelli H.M., Elgindy N. (2017). Hybrid Polymeric Matrices for Oral Modified Release of Desvenlafaxine Succinate Tablets. Saudi Pharm. J..

[26] Li L., Wang L., Li J., Jiang S., Wang Y., Zhang X., Ding J., Yu T., Mao S. (2014). Insights into the Mechanisms of Chitosan–Anionic Polymers-Based Matrix Tablets for Extended Drug Release. Int. J. Pharm..

[27] Samaro A., Vergaelen M., Purino M., Tigrine A., de la Rosa V.R., Goudarzi N.M., Boone M.N., Vanhoorne V., Hoogenboom R., Vervaet C. (2022). Poly (2-Alkyl-2-Oxazoline)s: A Polymer Platform to Sustain the Release from Tablets with a High Drug Loading. Mater. Today Bio.

[28] Gupta C.R., Kishore G.K., Ratna J.V. (2013). Development and Evaluation of Aceclofenac Matrix Tablets Using Polyethylene Oxides as Sustained Release Polymers. J. Pharm. Res..

[29] Grund J., Körber M., Bodmeier R. (2013). Predictability of Drug Release from Water-Insoluble Polymeric Matrix Tablets. Eur. J. Pharm. Biopharm..

[30] Knöös P., Schulz C., Piculell L., Ludwig R., Gorton L., Wahlgren M. (2014). Quantifying the Release of Lactose from Polymer Matrix Tablets with an Amperometric Biosensor Utilizing Cellobiose Dehydrogenase. Int. J. Pharm..

[31] Grund J., Koerber M., Walther M., Bodmeier R. (2014). The Effect of Polymer Properties on Direct Compression and Drug Release from Water-Insoluble Controlled Release Matrix Tablets. Int. J. Pharm..

[32] Agrawal S., Fernandes J., Shaikh F., Patel V. (2022). Quality Aspects in the Development of Pelletized Dosage Forms. Heliyon.

[33] Issa M.G., de Souza N.V., Duque M.D., Ferraz H.G. (2018). Physical Characterization of Multiparticulate Systems. Braz. J. Pharm. Sci..

[34] Alaimo A., Zhao X., Mi X., Atre P., Rizvi S.A.A. (2025). Advances in Oral Solid Drug Delivery Systems: Quality by Design Approach in Development of Controlled Release Tablets. BioChem.

[35] Singh K., Nainwal N., Chitme H.R. (2025). A Review on Recent Advancements in Pharmaceutical Technology Transfer of Tablets from an Indian Perspective. Ann. Pharm. Françaises.

[36] Prudhvi C., Sivaneswari S., Preethi N., Mounika B., Naveen Kumar B., Vasudeva Murthy S., Karthikeyan E. (2023). Development of Controlled-Release Matrix Tablets of Anti-Diabetic Agent Using Natural and Synthetic Polymers. Intell. Pharm..

[37] Viridén A., Wittgren B., Larsson A. (2009). Investigation of Critical Polymer Properties for Polymer Release and Swelling of HPMC Matrix Tablets. Eur. J. Pharm. Sci..

[38] Bagde S., Rohera B.D. (2024). Modification of the Swelling Behavior of a Hydrophilic Polymer as an Approach to Maintaining a Constant Gel Layer Thickness. J. Drug Deliv. Sci. Technol..

[39] Viridén A., Larsson A., Wittgren B. (2010). The Effect of Substitution Pattern of HPMC on Polymer Release from Matrix Tablets. Int. J. Pharm..

[40] Viridén A., Wittgren B., Andersson T., Larsson A. (2009). The Effect of Chemical Heterogeneity of HPMC on Polymer Release from Matrix Tablets. Eur. J. Pharm. Sci..

[41] Caviglioli G., Baldassari S., Cirrincione P., Russo E., Parodi B., Gatti P., Drava G. (2013). An Innovative Matrix Controlling Drug Delivery Produced by Thermal Treatment of DC Tablets Containing Polycarbophil and Ethylcellulose. Int. J. Pharm..

[42] Patil H., Tiwari R.V., Upadhye S.B., Vladyka R.S., Repka M.A. (2015). Formulation and Development of PH-Independent/Dependent Sustained Release Matrix Tablets of Ondansetron HCl by a Continuous Twin-Screw Melt Granulation Process. Int. J. Pharm..

[43] Verhoeven E., De Beer T.R.M., Schacht E., Van den Mooter G., Remon J.P., Vervaet C. (2009). Influence of Polyethylene Glycol/Polyethylene Oxide on the Release Characteristics of Sustained-Release Ethylcellulose Mini-Matrices Produced by Hot-Melt Extrusion: In Vitro and in Vivo Evaluations. Eur. J. Pharm. Biopharm..

[44] Saurí J., Zachariah M., Macovez R., Tamarit J.L., Millán D., Suñé-Pou M., García-Montoya E., Pérez-Lozano P., Miñarro M., Ticó J.R. (2017). Formulation and Characterization of Mucoadhesive Controlled Release Matrix Tablets of Captopril. J. Drug Deliv. Sci. Technol..

[45] Abdelbary G.A., Tadros M.I. (2008). Design and in Vitro/in Vivo Evaluation of Novel Nicorandil Extended Release Matrix Tablets Based on Hydrophilic Interpolymer Complexes and a Hydrophobic Waxy Polymer. Eur. J. Pharm. Biopharm..

[46] Verhoeven E., Vervaet C., Remon J.P. (2006). Xanthan Gum to Tailor Drug Release of Sustained-Release Ethylcellulose Mini-Matrices Prepared via Hot-Melt Extrusion: In Vitro and in Vivo Evaluation. Eur. J. Pharm. Biopharm..

[47] Muhamad H., Bashir A.B., Charlton-Harrison J., Abdulhussain R., Mawla N., Patel K., Williamson J., Blunt L., Walton K., Conway B. (2025). Hot-Melt Extruded-FDM 3D-Printed Polyethylene Oxide Tablets: Dissolution Imaging Analysis of Swelling and Drug Release. Eur. J. Pharm. Biopharm..

[48] Muhamad H., Ward A., Patel K., Williamson J., Blunt L., Conway B., Østergaard J., Asare-Addo K. (2024). Investigation into the Swelling and Dissolution Behaviour of Polymer-Excipient Blends of PEO Utilising Dissolution Imaging. Int. J. Pharm..

[49] Petrović J., Ibrić S., Betz G., Parojčić J., Durić Z. (2009). Application of Dynamic Neural Networks in the Modeling of Drug Release from Polyethylene Oxide Matrix Tablets. Eur. J. Pharm. Sci..

[50] Muhamad H., Mawla N., Dereiah S., Ward A., Williamson J., Asare-Addo K. (2024). Comparative Analysis of Drug Release Kinetics in Polyethylene Oxide and Xanthan Gum Matrices with Various Excipients. RSC Pharm..

[51] Heng P.W.S., Chan L.W., Easterbrook M.G., Li X. (2001). Investigation of the Influence of Mean HPMC Particle Size and Number of Polymer Particles on the Release of Aspirin from Swellable Hydrophilic Matrix Tablets. J. Control. Release.

[52] Sung K.C., Nixon P.R., Skoug J.W., Ju T.R., Gao P., Topp E.M., Patel M.V. (1996). Effect of Formulation Variables on Drug and Polymer Release from HPMC-Based Matrix Tablets. Int. J. Pharm..

[53] Barra J., Falson-Rieg F., Doelker E. (2000). Modified Drug Release from Inert Matrix Tablets Prepared from Formulations of Identical Composition but Different Organisations. J. Control. Release.

[54] Adeleke O.A. (2019). Premium Ethylcellulose Polymer Based Architectures at Work in Drug Delivery. Int. J. Pharm. X.

[55] Siepmann J., Kranz H., Bodmeier R., Peppas N.A. (1999). HPMC-Matrices for Controlled Drug Delivery: A New Model Combining Diffusion, Swelling, and Dissolution Mechanisms and Predicting the Release Kinetics. Pharm. Res..

[56] Saša B., Odon P., Stane S., Julijana K. (2006). Analysis of Surface Properties of Cellulose Ethers and Drug Release from Their Matrix Tablets. Eur. J. Pharm. Sci..

[57] Katakam P., Padala N.R., Chandu B.R., Elfituri A., Adiki S.K., Kommu R. (2013). Design of Lamivudine XR Matrix Tablets: Influence of HPMC and PEO on in Vitro Drug Release and Bioavailability in Rabbits. J. Pharm. Res..

[58] Saker A., Cares-Pacheco M.G., Marchal P., Falk V. (2019). Powders Flowability Assessment in Granular Compaction: What about the Consistency of Hausner Ratio?. Powder Technol..

[59] Kaleem M.A., Alam M.Z., Khan M., Jaffery S.H.I., Rashid B. (2021). An Experimental Investigation on Accuracy of Hausner Ratio and Carr Index of Powders in Additive Manufacturing Processes. Metal. Powder Report..

[60] Kawakita K., Lüdde K.H. (1971). Some Considerations on Powder Compression Equations. Powder Technol..

[61] Ilić I., Kása P., Dreu R., Pintye-Hódi K., Srčič S. (2009). The Compressibility and Compactibility of Different Types of Lactose. Drug Dev. Ind. Pharm..

[62] Alderborn G., Nyström C. (1996). Pharmaceutical Powder Compaction Technology.

[63] Fell J.T., Newton J.M. (2011). The Tensile Strength of Lactose Tablets. J. Pharm. Pharmacol..

[64] Farmacopea de los Estados Unidos de América USP Monographs Galantamina, Cápsulas de Liberación Prolongada. https://doi.usp.org/USPNF/USPNF_M5070_07_02.html.

[65] Yarce C.J., Pineda D., Correa C.E., Salamanca C.H. (2016). Relationship between Surface Properties and In Vitro Drug Release from a Compressed Matrix Containing an Amphiphilic Polymer Material. Pharmaceuticals.

[66] Švecová M., Palounek D., Volochanskyi O., Prokopec V. (2020). Vibrational Spectroscopic Study of Selected Alkaloids with Therapeutic Effects. Spectrochim. Acta A Mol. Biomol. Spectrosc..

[67] Valor A., Reguera E., Torres-García E., Mendoza S., Sanchez-Sinencio F. (2002). Thermal Decomposition of the Calcium Salts of Several Carboxylic Acids. Thermochim. Acta.

[68] Georgieva D., Nikolova D., Vassileva E., Kostova B. (2023). Chitosan-Based Nanoparticles for Targeted Nasal Galantamine Delivery as a Promising Tool in Alzheimer’s Disease Therapy. Pharmaceutics.

[69] Nagajothi A.J., Kannan R., Rajashabala S. (2018). Preparation and Characterization of PEO-Based Composite Gel-Polymer Electrolytes Complexed with Lithium Trifluoro Methane Sulfonate. Mater. Sci.-Pol..

[70] Babanejad N., Kandalam U., Omidi Y., Omidian H. (2022). Functional Properties of Thermally Tampered Poly (Ethylene Oxide). Bioimpacts.

[71] Zupanc A., Petkovšek M., Zdovc B., Žagar E., Zupanc M. (2024). Degradation of Hydroxypropyl Methylcellulose (HPMC) by Acoustic and Hydrodynamic Cavitation. Ultrason. Sonochem..

[72] Jani R., Patel D. (2015). Hot Melt Extrusion: An Industrially Feasible Approach for Casting Orodispersible Film. Asian J. Pharm. Sci..

[73] Gieroba B., Kalisz G., Krysa M., Khalavka M., Przekora A. (2023). Application of Vibrational Spectroscopic Techniques in the Study of the Natural Polysaccharides and Their Cross-Linking Process. Int. J. Mol. Sci..

[74] The Dow Chemical Company Ethylcellulose Polymers Tecnical Handbook. https://cms.chempoint.com/ic/getattachment/18c67e2f-5ae1-4989-97a4-23faf2cfd6ec/attachment.aspx.

[75] Dragar Č., Roškar R., Kocbek P. (2024). The Incorporated Drug Affects the Properties of Hydrophilic Nanofibers. Nanomaterials.

[76] Wong R.S.H., Dodou K. (2017). Effect of Drug Loading Method and Drug Physicochemical Properties on the Material and Drug Release Properties of Poly (Ethylene Oxide) Hydrogels for Transdermal Delivery. Polymers.

[77] Latreche M., Willart J.F. (2023). Analysis of the Dissolution Mechanism of Drugs into Polymers: The Case of the PVP/Sulindac System. Pharmaceutics.

[78] Fule R., Amin P. (2014). Hot Melt Extruded Amorphous Solid Dispersion of Posaconazole with Improved Bioavailability: Investigating Drug-Polymer Miscibility with Advanced Characterisation. Biomed. Res. Int..

[79] Anderson J.A., Lamichhane S., Vierhout T., Sherman A., Engebretson D., Pohlson K., Remund T., Kelly P. (2018). In Vitro Particulate and in Vivo Drug Retention Study of a Novel Polyethylene Oxide Formulation for Drug-Coated Balloons. J. Vasc. Surg..

[80] Crowley M.M., Schroeder B., Fredersdorf A., Obara S., Talarico M., Kucera S., McGinity J.W. (2004). Physicochemical Properties and Mechanism of Drug Release from Ethyl Cellulose Matrix Tablets Prepared by Direct Compression and Hot-Melt Extrusion. Int. J. Pharm..

[81] Bashir S., Zafar N., Lebaz N., Mahmood A., Elaissari A. (2020). Hydroxypropyl Methylcellulose-Based Hydrogel Copolymeric for Controlled Delivery of Galantamine Hydrobromide in Dementia. Processes.

[82] Li J., Zhao J., Tao L., Wang J., Waknis V., Pan D., Hubert M., Raghavan K., Patel J. (2015). The Effect of Polymeric Excipients on the Physical Properties and Performance of Amorphous Dispersions: Part I, Free Volume and Glass Transition. Pharm. Res..

[83] Ganjoo R., Soni S., Ram V., Verma A. (2016). Medium Molecular Weight Chitosan as a Carrier for Delivery of Lincomycin Hydrochloride from Intra-Pocket Dental Film: Design, Development, in Vitro and Ex Vivo Characterization. J. Appl. Pharm. Sci..

[84] Yasin H., Al-Taani B., Salem M. (2021). Preparation and Characterization of Ethylcellulose Microspheres for Sustained-Release of Pregabalin. Res. Pharm. Sci..

[85] Shah R.B., Tawakkul M.A., Khan M.A. (2008). Comparative Evaluation of Flow for Pharmaceutical Powders and Granules. AAPS PharmSciTech.

[86] Shojaee S., Emami P., Mahmood A., Rowaiye Y., Dukulay A., Kaialy W., Cumming I., Nokhodchi A. (2015). An Investigation on the Effect of Polyethylene Oxide Concentration and Particle Size in Modulating Theophylline Release from Tablet Matrices. AAPS PharmSciTech.

[87] Jeong G.Y., Bak J.H., Yoo B. (2019). Physical and Rheological Properties of Xanthan Gum Agglomerated in Fluidized Bed: Effect of HPMC as a Binder. Int. J. Biol. Macromol..

[88] Agrawal A.M., Manek R.V., Kolling W.M., Neau S.H. (2003). Studies on the Interaction of Water with Ethylcellulose: Effect of Polymer Particle Size. AAPS PharmSciTech.

[89] Huang W., Shi Y., Wang C., Yu K., Sun F., Li Y. (2013). Using Spray-Dried Lactose Monohydrate in Wet Granulation Method for a Low-Dose Oral Formulation of a Paliperidone Derivative. Powder Technol..

[90] Hu X., Xu T., Bu J., Cheng L., Wang T. (2025). Impact of Particle Size Distribution and Morphology of Starch on Microstructural–Functional Changes of Dough. LWT.

[91] Alyami H., Dahmash E., Bowen J., Mohammed A.R. (2017). An Investigation into the Effects of Excipient Particle Size, Blending Techniques and Processing Parameters on the Homogeneity and Content Uniformity of a Blend Containing Low-Dose Model Drug. PLoS ONE.

[92] Denduyver P., Vervaet C., Vanhoorne V. (2025). The Effect of Filler Particle Size on API Homogeneity of Controlled Release Formulations via Continuous Twin-Screw Wet Granulation. Int. J. Pharm..

[93] Jakubowska E., Ciepluch N. (2021). Blend Segregation in Tablets Manufacturing and Its Effect on Drug Content Uniformity—A Review. Pharmaceutics.

[94] United States Pharmacopeia 〈1174〉 (2023). Powder Flow.

[95] Rojas J., Uribe Y., Zuluaga A., Rojas J. (2012). Powder and Compaction Characteristics of Pregelatinized Starches. Pharmazie.

[96] Castañeda Hernández O., Domínguez-Robles J., Caraballo I., Bernad M.J., Melgoza Contreras L.M. (2023). Comparison between Polymeric Excipients Using SeDeM Expert System in Combination with Mathematical Modeling and Quality Control Tools. J. Drug Deliv. Sci. Technol..

[97] Kar A.K., Mahanti B., Kar B., Jana A., Chakrabarty S., Singh S., Majumdar S. (2025). Development, Optimization, and in-Vivo Bioavailability Study of Erlotinib Hydrochloride Loaded Microsponge for Colon Targeting. Intell. Pharm..

[98] Ren M., Xu H., Zhang X., Guan J., Mao S. (2024). Evaluation Methods and Strategies to Improve Compression Characteristics of Pharmaceutical Powders. J. Drug Deliv. Sci. Technol..

[99] Rashid I., Haddadin R.R., Alkafaween A.A., Alkaraki R.N., Alkasasbeh R.M. (2022). Understanding the Implication of Kawakita Model Parameters Using In-Die Force-Displacement Curve Analysis for Compacted and Non-Compacted API Powders. AAPS Open.

[100] Mishra S.M., Rohera B.D. (2019). Mechanics of tablet formation: A comparative evaluation of percolation theory with classical concepts. Pharm. Dev. Technol..

[101] Kuentz M., Leuenberger H. (2000). A New Theoretical Approach to Tablet Strength of a Binary Mixture Consisting of a Well and a Poorly Compactable Substance. Eur. J. Pharm. Biopharm..

[102] Mallick S., Kumar Pradhan S., Chandran M., Acharya M., Digdarsini T., Mohapatra R. (2011). Study of particle rearrangement, compression behavior and dissolution properties after melt dispersion of ibuprofen, Avicel and Aerosil. Results Pharma Sci..

[103] Grumann H.D., Kleinebudde P. (2023). Effect of Tableting Temperature on Tablet Properties and Dissolution Behavior of Heat Sensitive Formulations. Int. J. Pharm..

[104] Goldoozian S., Mohylyuk V., Dashevskiy A., Bodmeier R. (2021). Gel Strength of Hydrophilic Matrix Tablets in Terms of In Vitro Robustness. Pharm. Res..

[105] Adetunji O.A., Odeniyi M.A., Itiola O.A. (2015). Effect of Formulation and Process Variables on the Release, Mechanical and Mucoadhesive Properties of Ibuprofen Tablet Formulations. Acta Pol. Pharm. ñ Drug Res..

[106] Chandran S., Asghar L., Mantha N. (2008). Design and Evaluation of Ethyl Cellulose Based Matrix Tablets of Ibuprofen with PH Modulated Release Kinetics. Indian. J. Pharm. Sci..

[107] Verma R.K., Krishna D.M., Garg S. (2002). Formulation Aspects in the Development of Osmotically Controlled Oral Drug Delivery Systems. J. Control. Release.

[108] Huang X., Brazel C.S. (2001). On the Importance and Mechanisms of Burst Release in Matrix-Controlled Drug Delivery Systems. J. Control. Release.

[109] Zhang F., McGinity J.W. (2000). Properties of Hot-Melt Extruded Theophylline Tablets Containing Poly (Vinyl Acetate). Drug Dev. Ind. Pharm..

[110] Shaikh N.A., Abidi S.E., Block L.H. (1987). Evaluation of Ethylcellulose as a Matrix for Prolonged Release Formulations. I. Water Soluble Drugs: Acetaminophen and Theophylline. Drug Dev. Ind. Pharm..

[111] Hu A., Chen C., Mantle M.D., Wolf B., Gladden L.F., Rajabi-Siahboomi A., Missaghi S., Mason L., Melia C.D. (2017). The Properties of HPMC: PEO Extended Release Hydrophilic Matrices and Their Response to Ionic Environments. Pharm. Res..

[112] Seltzer B. (2010). Galantamine-ER for the Treatment of Mild-to-Moderate Alzheimer’s Disease. Clin. Interv. Aging.

