# Supplementary material for: Preformulation Study of Controlled-Release Galantamine Matrix Tablets Containing Polyethylene Oxide, Hydroxypropyl Methylcellulose, and Ethylcellulose
Source: Pharmaceutics. 2025 Aug 30;17(9):1139. doi: 10.3390/pharmaceutics17091139 (PMC12473912; doi:10.3390/pharmaceutics17091139)
Supplement: Supplementary file 1 [file pharmaceutics-17-01139-s001.zip › pharmaceutics-3794842-supplementary.pdf]

SUPPLEMENTARY MATERIAL

Particle size distribution parameters for individual ingredients and their corresponding formulation blends.

GAL

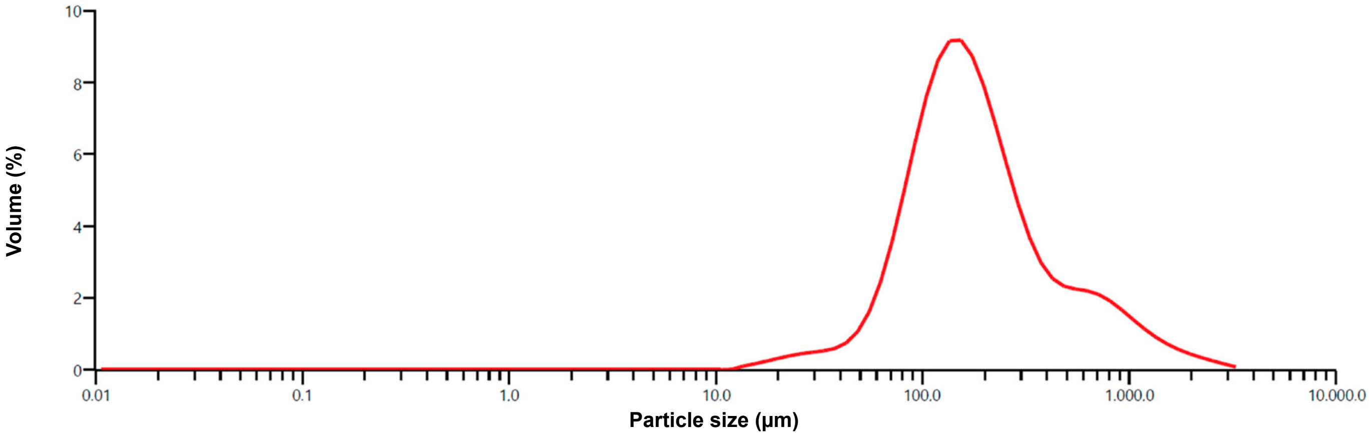

Figure S1. Particle size distribution of galantamine hydrobromide (GAL) measured by laser diffraction, shown as volume percentage versus particle size (μm).

PEO

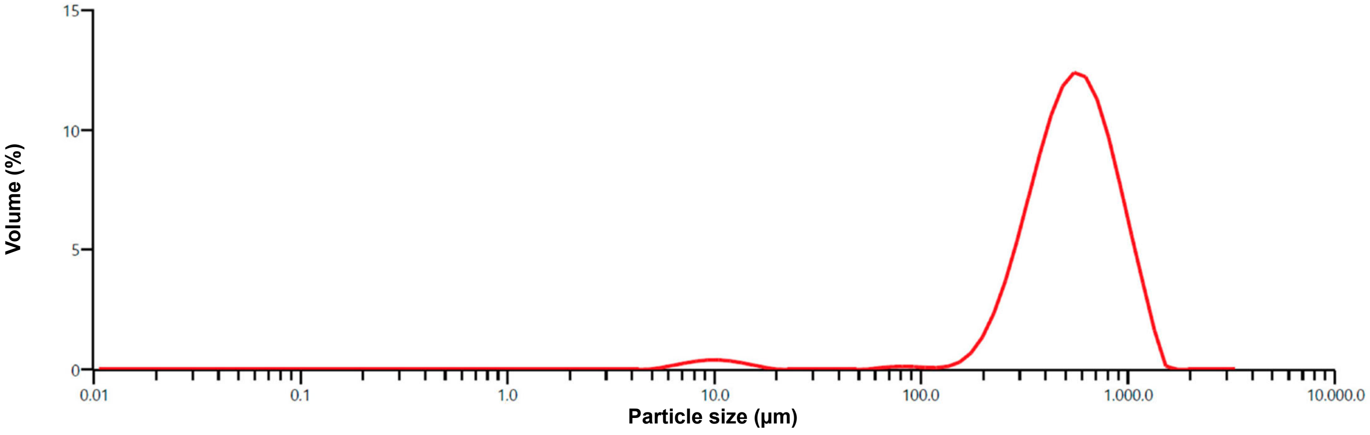

Figure S2. Particle size distribution of polyethylene oxide (PEO), presented as volume percentage versus particle size (μm).

HPMC

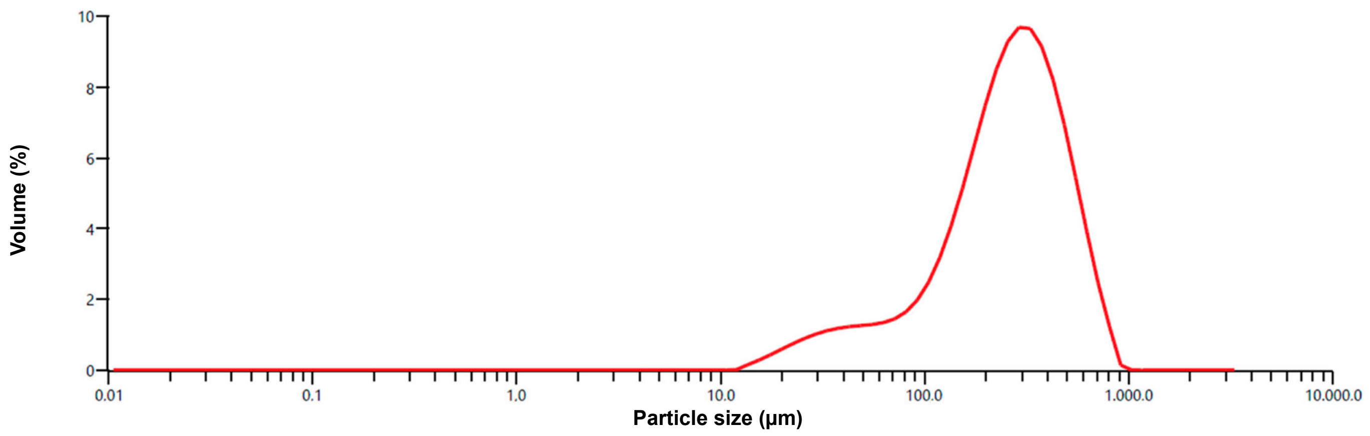

Figure S3. Particle size distribution of hydroxypropyl methylcellulose (HPMC), shown as volume percentage versus particle size (μm).

EC

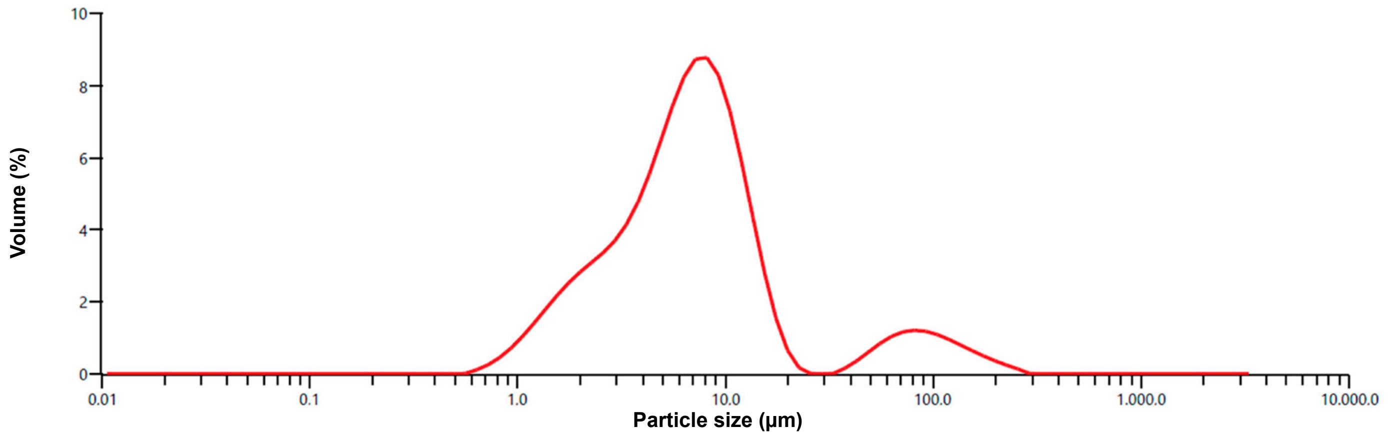

Figure S4. Particle size distribution of ethylcellulose (EC), expressed as volume percentage versus particle size (μm).

STARCH® 1500

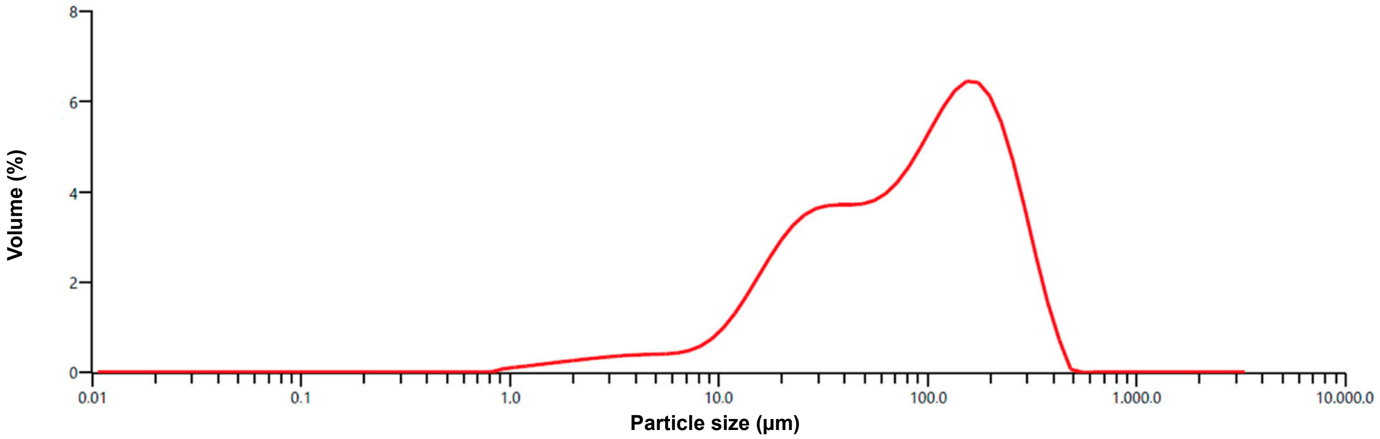

Figure S5. Particle size distribution of STARCH® 1500, represented as volume percentage versus particle size (µm).

SPRAY-DRIED LACTOSE MONOHYDRATE

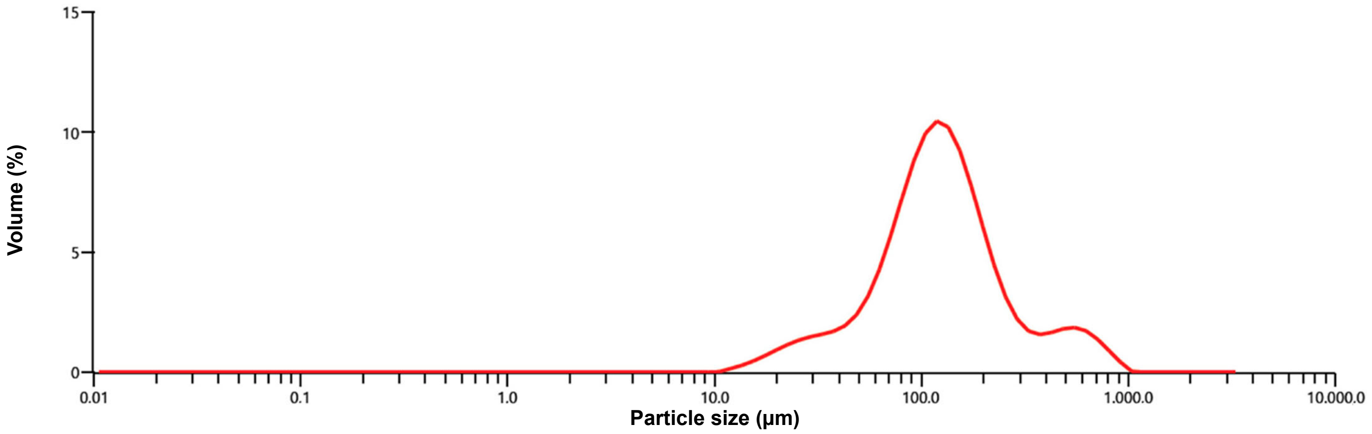

Figure S6. Particle size distribution of spray-dried lactose monohydrate, presented as volume percentage versus particle size (µm).

FORMULATION 1

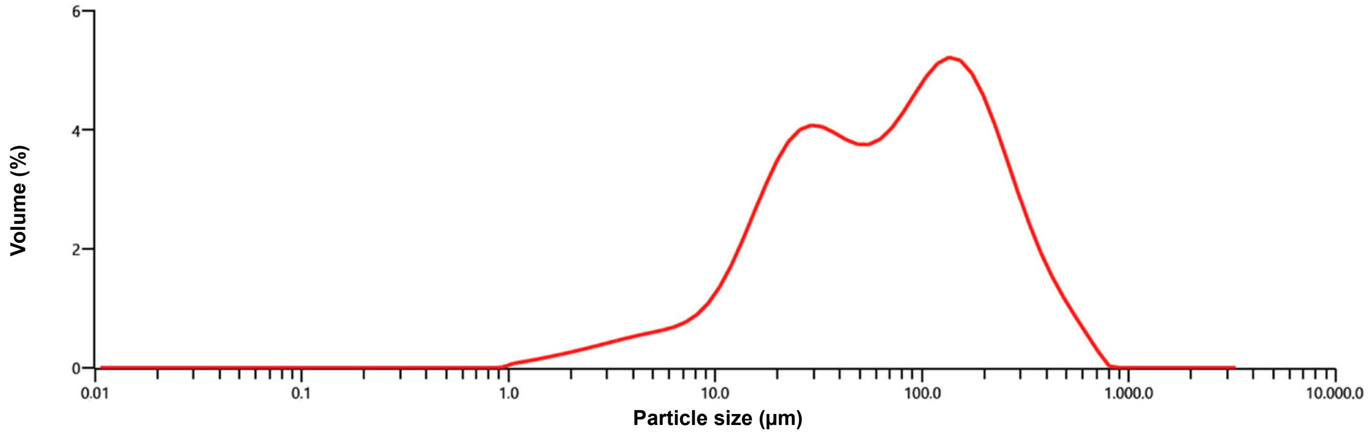

Figure S7. Particle size distribution of Formulation 1, showing volume percentage versus particle size (µm).

FORMULATION 2

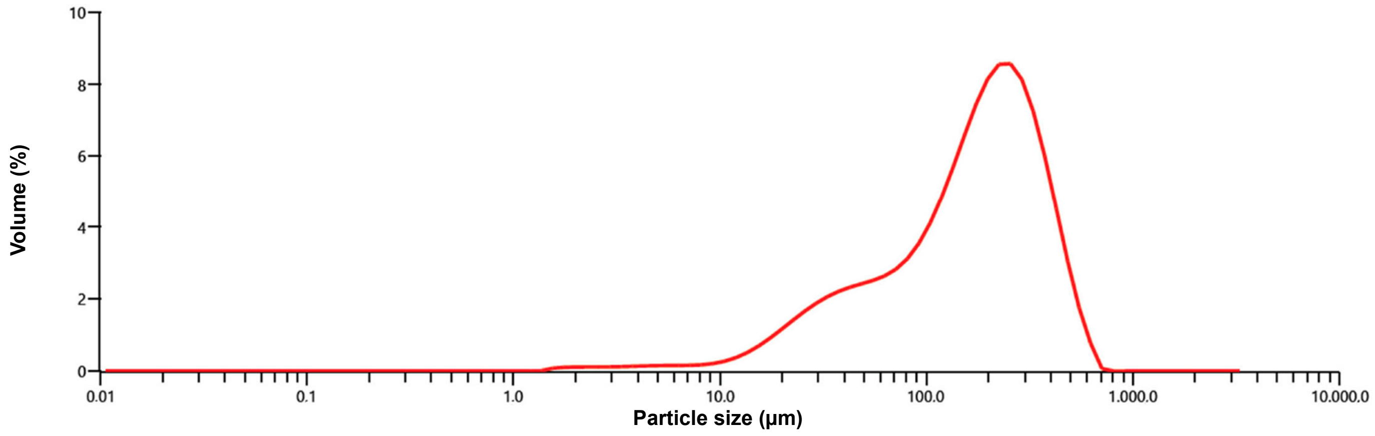

Figure S8. Particle size distribution of Formulation 2, showing volume percentage versus particle size (µm).

FORMULATION 3

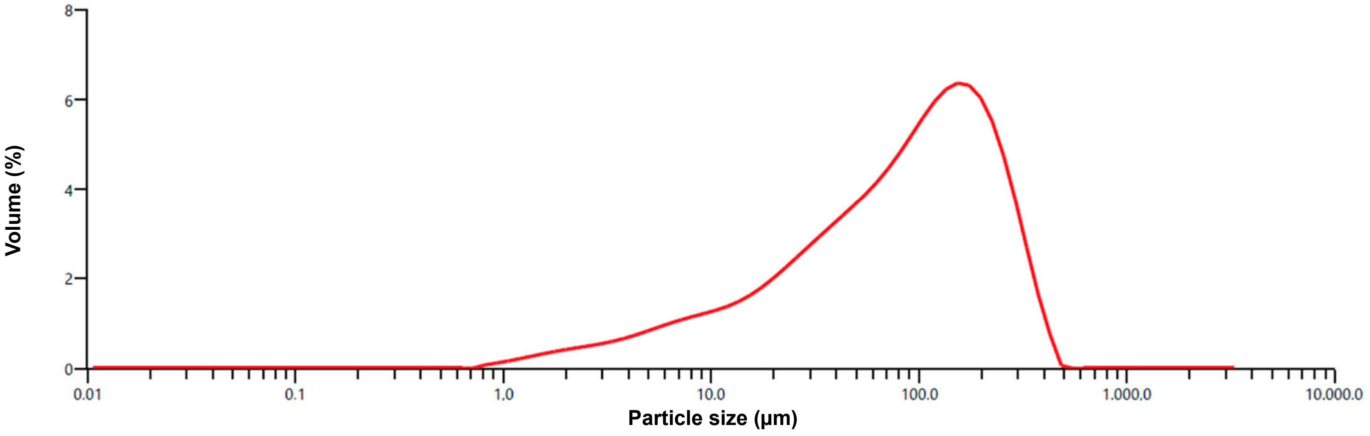

Figure S9. Particle size distribution of Formulation 3, showing volume percentage versus particle size (µm).
